# Phosphorylation-dependent modulation of CFTR macromolecular signalling complex activity by cigarette smoke condensate in airway epithelia

**DOI:** 10.1038/s41598-019-48971-y

**Published:** 2019-09-03

**Authors:** Andrea Schnúr, Aiswarya Premchandar, Miklos Bagdany, Gergely L. Lukacs

**Affiliations:** 1 Department of Physiology, McGill University, Montréal, Quebec Canada; 2 Department of Biochemistry, McGill University, Montréal, Quebec Canada

**Keywords:** Chloride channels, Ion transport, Mechanisms of disease, Phosphorylation

## Abstract

Genetic and acquired loss-of-function defect of the cystic fibrosis transmembrane conductance regulator (CFTR) compromise airway surface liquid homeostasis and mucociliary clearance (MCC), culminating in recurrent lung inflammation/infection. While chronic cigarette smoke (CS), CS extract (CSE; water-soluble compounds) and CS condensate (CSC; particulate, organic fraction) exposure inhibit CFTR activity at transcriptional, biochemical, and functional levels, the acute impact of CSC remains incompletely understood. We report that CSC transiently activates CFTR chloride secretion in airway epithelia. The comparable CFTR phospho-occupancy after CSC- and forskolin-exposure, determined by affinity-enriched tandem mass spectrometry and pharmacology, suggest that localised cAMP-dependent protein kinase (PKA) stimulation by CSC causes the channel opening. Due to the inhibition of the MRP4/ABCC4, a cAMP-exporter confined to the CFTR macromolecular signalling-complex, PKA activation is accomplished by the subcompartmentalised elevation of cytosolic cAMP. In line, MRP4 inhibition results in CFTR activation and phospho-occupancy similar to that by forskolin. In contrast, acute CSC exposure reversibly inhibits the phosphorylated CFTR both *in vivo* and in phospholipid bilayers, without altering its cell surface density and phospho-occupancy. We propose that components of CSC elicit both a transient protective CFTR activation, as well as subsequent channel block in airway epithelia, contributing to the subacute MCC defect in acquired CF lung diseases.

## Introduction

The adverse long-term effects of inhaled, combusted tobacco or cigarette smoke (CS) on the lung’s cellular and molecular physiology have been established and include DNA damage, goblet cell metaplasia, increased inflammation, autophagy and proteolysis^1^. These processes account for higher incidence of chronic obstructive pulmonary disease (COPD), the third leading cause of death in the US^2^, which is compounded by significant extrapulmonary pathologies^1,3^ and lung cancer. The acquired loss of the cystic fibrosis transmembrane conductance regulator (CFTR) function, a chloride and bicarbonate-selective, 3′,5′-cyclic adenosine monophosphate (cAMP)-dependent protein kinase (PKA) regulated anion channel^4^, is invoked in the pathogenesis of COPD partly as a consequence of CS exposure^5,6^. COPD exhibits hallmarks of chronic bronchitis and emphysema, with overlapping clinical manifestation and molecular pathology to that of cystic fibrosis (CF)^7,8^. Both acquired and inherited expression defects of CFTR result in the periciliary liquid layer depletion, acidification, mucus dehydration, increased bacterial adhesion, and decreased mucociliary clearance (MCC), causing recurrent infections and sustained inflammation with progressive deterioration of the lung tissue^6,9–12^. Compelling evidence indicates that subacute (2–24 h) and chronic (>24 h) cigarette smoke (CS) exposure compromises CFTR activity at the protein and mRNA level in a variety of cell types, including human nasal and bronchial epithelia^11,13–20^. In contrast, the acute effect of CS on CFTR is incompletely understood.

CFTR, a member of the adenosine triphosphate (ATP)-binding cassette transporter (ABC) superfamily, is predominantly localised at the apical plasma membrane (PM) of secretory and resorptive epithelia in various organs, including the lung, intestine, pancreas, sweat ducts, and vas deferens^21,22^. CFTR, in concert with the epithelial Na^+^ channel (ENaC) and other transporters (e.g. calcium-activated chloride channel - TMEM16A, anion exchangers of the SLC26A family, sodium-potassium-chloride cotransporter (NKCC1), sodium-bicarbonate cotransporters (NBC1), chloride-bicarbonate exchanger (AE2)), regulate the transepithelial ion and water movement and, thereby, the airway surface liquid (ASL) homeostasis^23,24^.

The PKA-dependent phosphorylation of the regulatory domain (RD) is required to suspend the pore blockage and to permit the ATP-dependent heterodimerization of the nucleotide-binding domains 1 and 2 (NBD1-NBD2), initiating mechano-chemical coupling of CFTR pore opening^25,26^. The phosphorylation-dependent CFTR activity is fine-tuned by the macromolecular signalling complex of CFTR at the apical PM of airway epithelia^27,28^. This complex consists of kinases (e.g. PKA, AMPK), phosphatases (e.g. PP2A), adenylyl cyclases (e.g. AC1), phosphodiesterases (e.g. PDE4 and PDE3A) that either directly or indirectly *via* scaffolding proteins (e.g. actin, NHERF1/2 and PDZK1) are associated with CFTR^28^. The localized cAMP concentration in the vicinity of the channel, is also influenced by multidrug resistance-associated proteins, e.g. the multidrug resistance-associated protein 4 (MRP4) activity, an ABC transporter that can extrude cytosolic cAMP^29–31^. This complex arrangement fine tunes the spatio-temporal regulation of CFTR phosphorylation-dephosphorylation events^28^. Biochemical and spectroscopic techniques established that ten known Ser/Thr PKA consensus sites and five additional phosphosites had to be mutated in the RD and the NBD1 to completely inactivate the channel (15SA-CFTR) in Chinese hamster ovary (CHO) cells^32^. However, the phospho-occupancy under resting and stimulated conditions has been determined only for the S660 and S737 PKA consensus sites in CFTR expressed in baby hamster kidney (BHK) cells upon PKA activation^33^.

The effect of CS, containing over 4,000 constituents, on the CFTR functional expression can be modelled by exposing cells to whole CS, or solutions that absorbed the water- or organic solvent-soluble components of CS. CS extract (CSE) is obtained by dissolving the water-soluble gas and particulate phase of the CS, partially capturing the complexity of the CS. In contrast, CS condensate (CSC) is prepared by dissolving the particulate phase of CS in an organic solvent^34^. Acutely, CSE has been shown to inhibit chloride secretion in the canine trachea, *Xenopus* oocyte, and human bronchial epithelial cells (HBE)^13,15,16^. More recently, the transient activation of the CFTR I_sc_ by CSE was documented in CFBE and primary HBE and proposed as a defensive mechanism against the accumulation of toxic compounds in the ASL^35^. Considering the compositional differences of CSE and CSC^34^, and observations that documented only CFTR inhibition by the acute CSC exposure of murine and human nasal epithelia^13,14^, we set out to examine the acute impact of CSC on CFTR in human bronchial epithelia (HBE). Here, we report that CSC elicits a robust CFTR-mediated anion secretion, a mechanism distinct from that of CSE in HBE, which is followed by the reversible inhibition of the channel, documented *in vivo* and *in vitro*. We uncovered that inhibition of MRP4 activity by the CSC accounts, at least partly, for the localised cAMP elevation and coupled PKA activation in the vicinity of CFTR. In support of this mechanism, the phospho-occupancy of CFTR, established by a label-free, targeted quantitation approach involving Electron-Transfer and Higher-Energy Collision Dissociation (EThcD) tandem mass spectrometry (MS/MS) of affinity-enriched CFTR showed similar patterns in the presence of CSC, MRP4 inhibition, and PKA activation by forskolin.

## Results

### Cigarette smoke condensate acutely stimulates CFTR function in airway epithelia

We examined the acute effect of CSC on the wild-type (WT) CFTR activity in CFBE41o- (CFBE), a human cystic fibrosis bronchial epithelial cell line^36^ with the *CFTR*^*ΔF508/ΔF508*^ genotype but no detectable endogenous CFTR protein expression^37^. We transduced CFBE with lentivirus encoding the wild-type (WT) CFTR, harbouring a triple hemagglutinin (3HA) epitope tag in its 4^th^ extracellular loop and expressed under the control of the tetracycline-responsive transactivator^38^. Filter-grown CFBE were differentiated for at least four days post-confluence, while CFTR expression was adjusted to be less than the endogenous level in Calu-3 epithelia, induced with 50–250 ng/ml doxycycline^38^.

**Figure 1:**
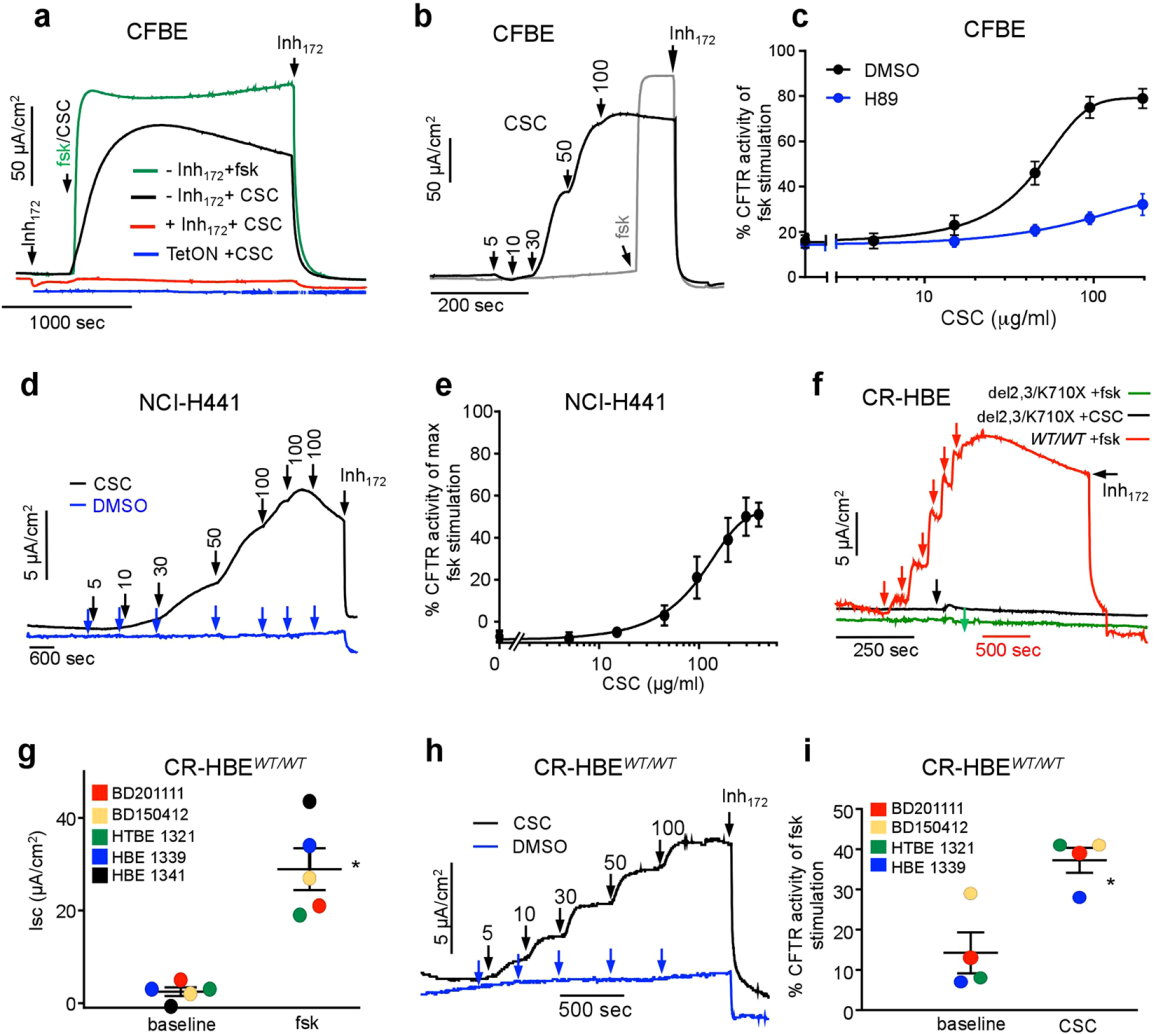
Cigarette smoke condensate (CSC) causes dose-dependent increase in CFTR mediated chloride secretion. (**a**) Representative short-circuit current (I_sc_) traces showing the effect of 10 μM forskolin (green) or 200 μg/ml CSC on CFTR expressing (Inh_172_, black), non-expressing (TetON, blue), and with 20 μM CFTR specific inhibitor, Inh_172_, pre-treated cells (+Inh_172_, red). All I_sc_ measurements with CFBE have been done after basolateral permeabilisation in the presence of a basolateral-to-apical chloride gradient and amiloride (100 μM) unless otherwise stated. (**b**) The effect of CSC on the CFTR-mediated I_sc_. Arrows represent sequential addition of CSC (5, 10, 30, 50, and 100 μg/ml, black) or forskolin (10 μM, gray) followed by Inh_172_. (**c**) The dose response curve of CSC without (black) and with H89 (blue) pre-treatment on the Inh-_172_-sensitive I_sc_ in WT-CFTR-expressing CFBE. Data are means ± SEM, n = 5. (**d**) The effect of CSC (black, added at the indicated μg/ml concentration) or DMSO (blue) followed by the 20 μM In_172_ on the I_sc_ in polarized NCI-H441 cells after basolateral permeabilization in the presence of a basolateral-to-apical chloride gradient. (**e)** Quantification of CSC effect on CFTR–mediated I_sc_ in NCI-H441 cells. Data represent mean ± SEM, n = 3 performed in technical duplicates. (**f**) Effect of CSC (200 μg/ml) or forskolin addition on CFTR ^WT/WT^ and the CFTR ^del2,3/ K710X^ truncation mutant expressing CR-HBE respectively. Arrows indicate the addition of 200 μg/ml CSC (black), 20 μM forskolin (green) or the sequential addition of 0.01, 0.04, 0.1, 0.2, 1–2, 10 μM forskolin (red). I_sc_ measurements were performed with equimolar chloride concentrations in both chambers, without basolateral permeabilisation. (**g**) Summary of maximal forskolin stimulation on CFTR-mediated *I*_*sc*_ in CR-HBE ^WT/WT^ derived from five individuals. Forskolin stimulation or basal current was calculated relative to baseline after Inh_172_. Each dot represents the average of 2–4 technical replicates per cell-line. *p < 0.01 *vs* baseline (**h**) CR-HBE ^WT/WT^ cells dose response to CSC (black), measured by I_sc_. CSC additions: 5, 10, 30, 50 and 100 μg/ml. (**i**) Summary of CSC (200 μg/ml) effect on CFTR activity in CR-HBE cells from four individuals. CSC stimulation or basal current was calculated relative to baseline after Inh_172_ and expressed as a percentage of maximal forskolin stimulation. Each dot represents the average of 2–4 technical replicates. *p < 0.01.

We determined the apical PM chloride conductance by short circuit current (I_sc_) measurements in the presence of a basolateral-to-apical chloride gradient after inhibiting the apical sodium channels (ENaC) with amiloride and permeabilising the basolateral PM with amphotericin B. Apically administered CSC (200 μg/ml) triggered a robust increase in the I_sc_ that peaked after ~10 min, followed by a gradual decline in I_sc_ (Fig. 1a). In contrast to the CSC effect, adenylyl cyclases (AC) activation by forskolin elicited a sustained CFTR I_sc_, implying that the forskolin-induced PKA activation remains resistant to inactivation throughout the measurement (Fig. 1a). Both the CSC- and forskolin-stimulated I_sc_ was abrogated by a CFTR-specific inhibitor (Inh_172_) and absent in TetON-CFBE cells that lack CFTR expression (Fig. 1a). According to the dose-response, the CSC EC_50_ was at ~50 μg/ml and the I_sc_ maximum was ~80% of that of the forskolin-stimulated peak current (Fig. 1b,c). H89, a potent inhibitor of PKA (EC50 ~135 nM)^39^, suppressed the CSC evoked CFTR I_sc_ by ~75% (Fig. 1c, blue trace), implying that CSC effect is exerted, predominantly, *via* PKA activation.

Considering the cell-type dependent responses to CSE^40^, we evaluated the effect of acute CSC administration in the polarised papillary lung adenocarcinoma cell line (NCI-H441), transduced with WT-CFTR expressing lentivirus. NCI-H441 cells displayed similar dose-dependent and Inh_172_-sensitive CFTR activation in the presence of CSC than the CFBE (Fig. 1d,e). The CSC EC_50_ was ~110 ± 26 μg/ml (±SEM, n = 3).

Next, the CSC effect was examined on conditionally reprogrammed-primary human bronchial epithelial cells (CR-HBE) derived from five non-CF individuals, following their differentiation at ALI, as described in Methods^41,42^. Robust, Inh_172_-sensitive I_sc_ was activated by forskolin (Fig. 1f,g), as well as by CSC exposure (Fig. 1h,i) with an EC_50_ of 38 ± 6.8 μg/ml (±SEM, n = 4) in CR-HBE^WT/WT^. To support the role of CFTR in I_sc_ stimulation in CR-HBE^WT/WT^, similar experiments were performed on CR-HBE encoding two non-functional CFTR alleles (del2,3/K710X)^43^. Neither CSC nor forskolin provoked measurable CFTR I_sc_ in CR-HBE^*del2*,*3/K710X*^ (Fig. 1f). These observations are consistent with the conclusion that the contribution of CFTR-independent chloride secretion is negligible to the acute I_sc_ activation by CSC in HBE.

### CFTR acute activation by CSC is independent of cellular oxidative stress

To dissect the PKA activation mechanism of CFTR, first, we considered whether reactive oxygen species (ROS) production contributes to the enhanced chloride secretion as described for the acute CSE effect^35^. CSE-induced oxidative stress stimulates PKA-dependent CFTR activation *via* prostanoids production and Ca^2+^-release from the ER^35,44–48^. We tested whether CSC can provoke oxidative stress by using the fluorescence intracellular ratiometric redox-sensor, Grx1-roGFP^49^, stably expressed in CFBE (Supplementary Fig. 1a). Exposure of CFBE to H_2_O_2_ augmented the fluorescence ratio at 405/488 nm, reflecting the Grx1-roGFP^50^ oxidation by H_2_O_2_ with an EC50 of 41 ± 6.8 μM (n = 4), which reached a maximum value at ~100 μM H_2_O_2_ (Supplementary Fig. 1b). Concomitantly, the CFTR I_sc_ was activated to near the forskolin-stimulated level and the intracellular cAMP level was increased by ~18 fold (from 8 ± 0.9 to 163 ± 53 pmol/ml, n = 2 (not shown) (Supplementary Fig. 1c,d).

If oxidative stress plays a determinant role in the acute CSC effect, reducing agents may prevent CFTR activation. This possibility was tested by examining the effect of glutathione monoethyl-ether (GSH-ME, 1 mM, 4 h), N-acetylcysteine (NAC, 5 mM, 4 h), or reduced L-glutathione (GSH, 10 mM, 10 min) preincubation, first on the H_2_O_2_-induced Grx1-roGFP oxidation and then on the I_sc_ in CFBE. These compounds suppressed the Grx1-roGFP oxidation by 30–90% (Supplementary Fig. 1c). GSH, which caused near complete prevention of the Grx1-roGFP oxidation, similar to the less efficient NAC, was unable to thwart the CSC-induced CFTR I_sc_ activation (Supplementary Fig. 1d,e). Jointly, these results suggest that CFTR activation by CSC is independently evoked of the cellular oxidative stress.

### CSC inhibits the phosphorylated CFTR activity in airway epithelia and phospholipid bilayer

**Figure 2:**
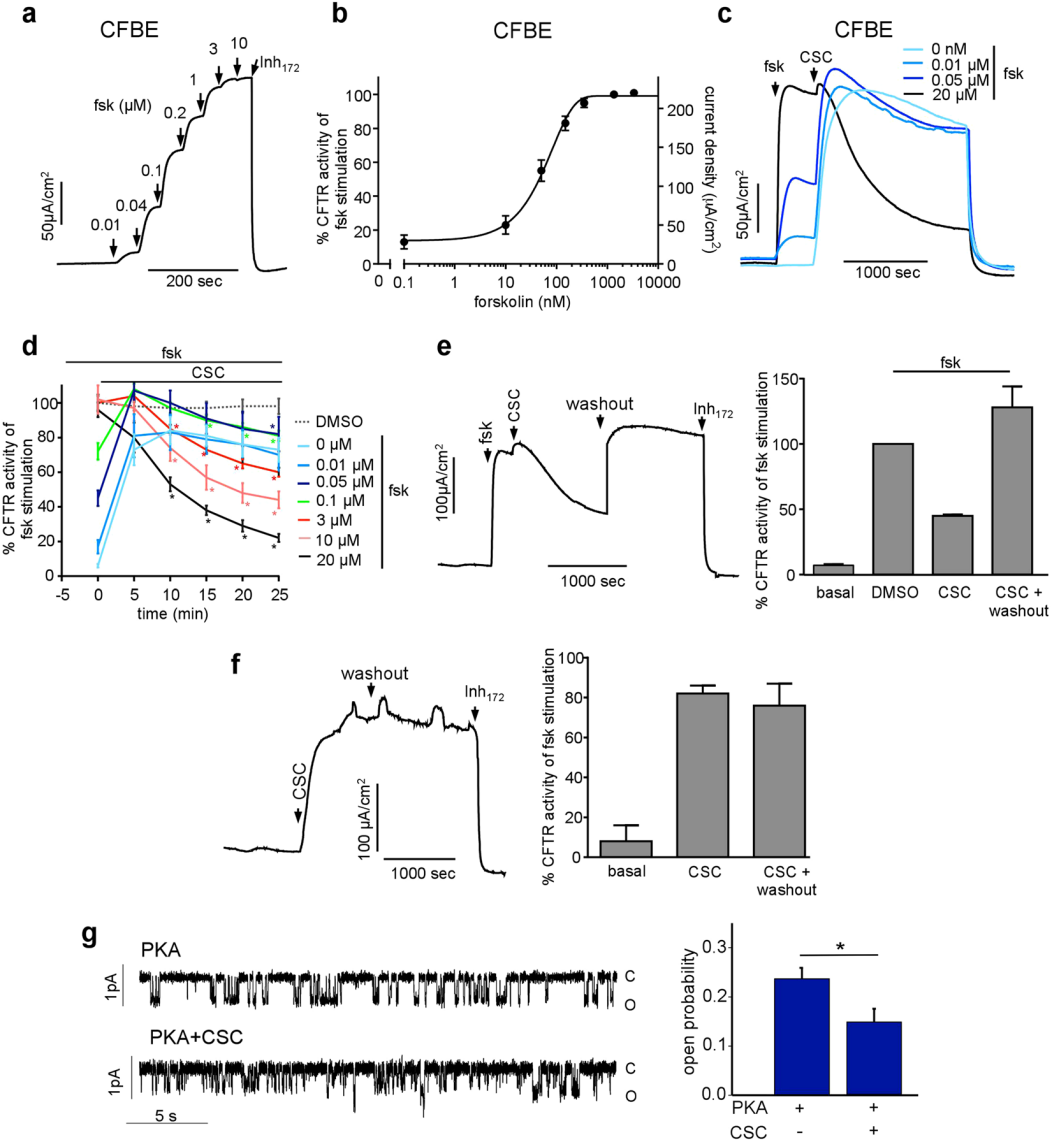
CSC inhibits the phosphorylated CFTR activity. (**a)** Representative *I*_*sc*_ recordings and (**b**) summary figure of forskolin dose-response in WT-CFTR expressing CFBE. Data are means ± SEM, n = 8. (**c**) Representative I_sc_ recording shows the CSC (200 μg/ml) effect on CFTR activity in the absence or following activation of the channel by the indicated concentration of forskolin in CFBE. (**d**) Summary of CSC (200 μg/ml) or DMSO (dotted line) effect on CFTR Cl^−^ transport, stimulated with the indicated concentration of forskolin in CFBE. Data are means ± SEM, n = 3–4 in duplicates. *p < 0.05 *vs* 5 min. (**e**) The inhibitory effect of 200 μg/ml CSC on CFTR activity in the presence of 20 μM forskolin could be washed out in CFBE. (**f**) CFTR activation by 200 μg/ml CSC in CFBE, measured by short-circuit current recording, cannot be reversed by washout. Right panels show the summary of CFTR current before and after washout in the presence or absence of forskolin calculated as % to maximal forskolin for panels, (**e**,**f**). Data represent the means of three biological replicates. (**g) **Single-channel recordings of phosphorylated CFTR channel in lipid bilayers in the absence (above) and presence (below) of CSC (200 μg/ml). Open (o) and closed (c) states are indicated (left panel). Mean open probabaility of phsophorylated CFTR in the absence and presence of CSC (200 μg/ml) (mean ± SEM, n = 16–18, right panel).

CFTR I_sc_ activation was not sustainable upon CSC stimulation, in contrast to that of forskolin in CFBE and CR-HBE (Fig. 1a and data not shown). This observation may reflect one or more of the following events: the PKA-activated channel’s increasing susceptibility to inhibitor(s), progressive intracellular accumulation/production of CFTR inhibitor(s), activation of phosphatases, and inhibition of PKA activity by CSC either directly or indirectly. To assess some of these possibilities, first, we tested the effect of CSC on CFTR at various activation levels in the presence of increasing forskolin concentrations (Fig. 2a,b). Partially activated CFTR was modestly susceptible to inhibition by CSC (Fig. 2c,d). At maximal CFTR activation, CSC caused a robust and fast inhibition of the channel (Fig. 2c,d). Thus, the onset and extent of CFTR I_sc_ inhibition by CSC may depend on the phosphorylation state of the channel (Fig. 2c,d). Importantly, the CSC-induced CFTR I_sc_ inhibition was rapidly and fully reversible by washing out the CSC (Fig. 2e). In contrast, CFTR activation by CSC was irreversible (Fig. 2f), suggesting distinct mechanisms of activation and inhibition by CSC.

To assess whether cytosolic proteins are required for CFTR inhibition by CSC, we tested the CSC effect on the channel activity after incorporation into planar phospholipid bilayers. CFTR-containing microsomes were isolated from BHK cells, *in vitro* phosphorylated by the PKA catalytic subunit, and then fused to the phospholipid bilayer in the absence of cytosolic proteins (e.g. phosphatases), as described^51^. CSC decreased the open probability (P_o_) of the activated CFTR by 48% from 0.24 ± 0.02 to 0.15 ± 0.03 (n = 16–18) in the phospholipid bilayer (Fig. 2g). This observation suggests that altered activity of cytosolic phosphatases, ACs or PKAs cannot explain the acute inhibitory effect of CSC on CFTR.

**Figure 3:**
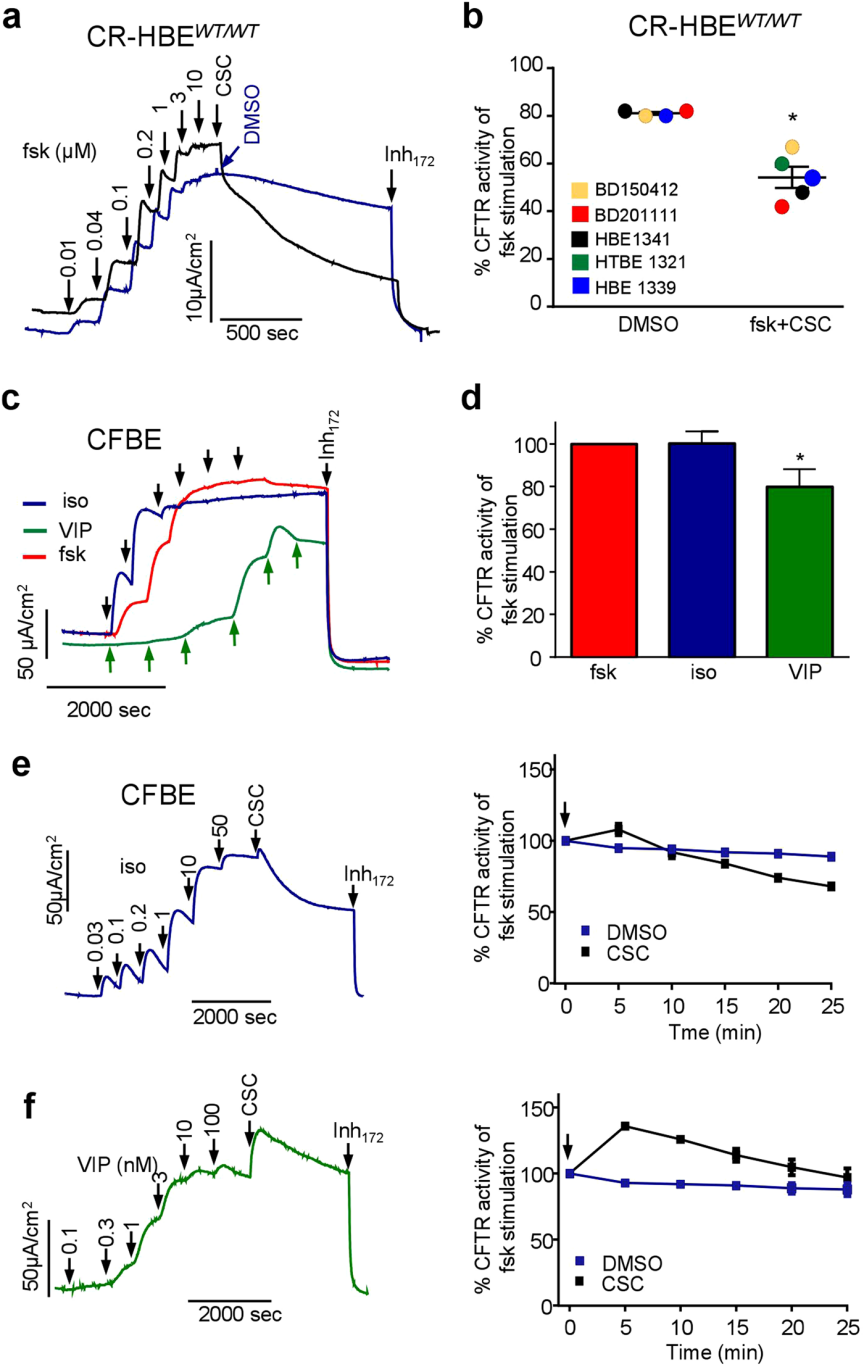
CSC inhibits the phosphorylated CFTR activity in CR-HBE and following ligand-induced activation of CFBE. (**a**) Effect of CSC (200 μg/ml) or DMSO on forskolin activated *I*_*sc*_ in CR-HBE^WT/WT^ cells. (**b)** Summary CSC (200 μg/ml) or DMSO effect after 20 min on forskolin-stimulated CFTR-mediated I_sc_. Each dot represents the average of 2–4 technical replicates per cell-line from five individuals. *p < 0.05 (**c**) Representative I_sc_ traces on CFBE after isoproterenol (Iso, delta concentration: 0.03, 0.1, 0.3, 1, and 10 μM), forskolin (0.01, 0.04, 0.1, 0.2, 1, 2, and 10 μM) or VIP (0.1, 0.3, 1.0, 3.0, 10, and 100 nM) stimulation. (**d)** Summary of maximal CFTR-mediated current stimulation by Isoproterenol (50 μM) and VIP (100 nM) compared to maximal forskolin stimulation (10 μM). Data are means ± SEM, n = 3 of technical duplicates *p < 0.05 (**e**) Representative *I*_*sc*_ traces and summary figures (right panels, n = 3 with duplicates) of the β-adrenoreceptor agonist, isoproterenol activation (upper panels) or (**f**) VIP stimulation followed by CSC (200 μg/ml, black line) or DMSO administration.

The CSC inhibitory effect was reproduced in CR-HBE following the channel activation by forskolin (Fig. 3a). CSC reduced the CFTR I_sc_ by ~40% within 25 min in CR-HBE, obtained from non-CF-individuals (Fig. 3b). The CSC inhibitory effect was examined upon CFTR stimulation by ß_2_-adrenergic or vasoactive intestinal peptide (VIP) receptors stimulation in CFBE. These receptors were exposed to the agonist isoproterenol or VIP, respectively, which generated 80–100% of the forskolin-provoked CFTR I_sc_ (Fig. 3c,d). Subsequent exposure of the cells to CSC inhibited the agonist-induced I_sc_ by ~30% within 25 min (Fig. 3e,f). These experiments suggest that the phosphorylated CFTR is susceptible to inhibition by CSC to variable extent in different cell types independent of the PKA activation pathway.

### Cell surface density of CFTR is preserved upon acute CSC exposure

CFTR apical cell surface density is regulated by exocytosis and endocytosis. Endocytic sequestration has been implicated in the removal of functional CFTR from the cell surface after CSE or CS incubation for 10 min to 120 hours^11,52–55^. We probed whether CFTR exocytosis or endocytosis contributes to the CSC acute activation or inhibition of CFTR I_sc_, respectively. The PM density of WT-CFTR-3HA was measured by PM ELISA or domain-specific biotinylation on filter-grown CFBE.

**Figure 4:**
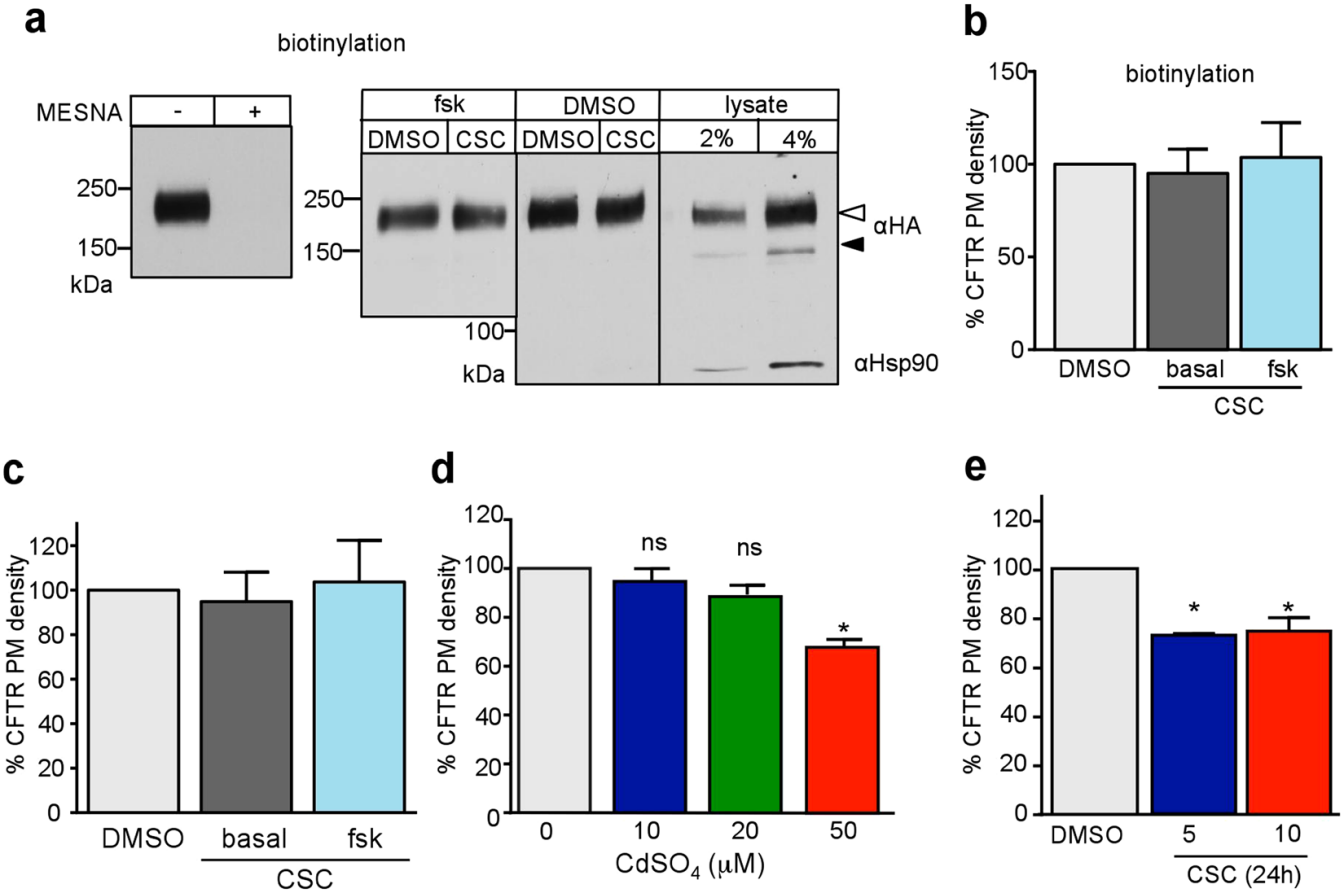
Acute and chronic effect of CSC on CFTR PM expression. (**a**) The effect of 30 min 200 μg/ml CSC on CFTR PM density in CFBE was measured by cell surface biotinylation with NHS-SS-biotin and immunoblotting of the affinity-purified biotinylated proteins. PM selectivity of biotinylation was demonstrated by complete reversibility of biotin-conjugation with the cell -impermeable reducing agent MESNA (50 mM, left panel). Neither the core-glycosylated CFTR nor cytosolic Hsp90 was susceptible to biotinylation, indicating that covalent labelling of the PM by biotin was selective to the cell surface proteins (right panel). Complex- and core-glycosylated CFTR are indicated by an empty and filled arrowhead, respectively. (**b)** Quantification of PM resident CFTR-3HA after 30 min of 200 μg/ml CSC treatment with or without 10 min of forskolin stimulation by the biotinylation assay as in panel b or Data are means n = 9–10 (**c**) CFTR detection by cell surface ELISA with Amplex red substrate. Data are means ± SEM, n = 3–6 (**d**) Chronic (24 h) CdSO_4_ or (**e)** CSC treatment in CFBE CFTR PM density measured by ELISA with Amplex red assay. Data are means ± SEM, n = 3, *p < 0.05.

CFBE was treated with CSC (200 μg/ml) or DMSO in Krebs solution (KRB) for 25 min at 37 °C, to mirror the condition of the I_sc_ measurements. Apical membrane proteins were labelled with NHS-SS-biotin on ice and CFTR was visualised after streptavidin affinity purification by immunoblotting (Fig. 4a). We could not resolve significant changes in the abundance of biotinylated CFTR following CSC exposure, either in the absence or presence of forskolin stimulation (Fig. 4b). Based on the following observations, only PM, but not intracellular proteins were susceptible to biotinylation: a) NHS-SS-biotin was completely cleaved from CFTR by the cell-impermeant reducing agent 2-mercaptoethanesulfonate (MESNA, Fig. 4a, left panel). b) Neither the Hsp90, a cytosolic molecular chaperone, nor the ER-resident core-glycosylated CFTR were susceptible to biotinylation but were readily detectable in the cell lysate (Fig. 4a, right panel).

To support the biotinylation results, CFTR PM density was monitored by PM ELISA, using a mouse primary anti-HA antibody (Ab) and a horseradish peroxidase (HRP)-conjugated secondary Ab. Specific anti-HA Ab binding was measured as described in Methods. Acute activation or inhibition of the channel by CSC or forskolin + CSC treatment did not result any significant changes in the CFTR expression at the apical PM expression of CFBE (Fig. 4c). Chronic exposure of CFBE to CSC (5–10 μg/ml) or to CdSO_4_ (50 μM), constituents of CS, however, significantly reduced the CFTR PM expression (Fig. 4d,e), confirming the biochemical downregulation of CFTR at the PM, as previously reported^20^.

### Compartmentalised activation of CFTR by CSC

CFTR gating primarily relies on PKA-dependent phosphorylation of the regulatory domain (RD)^56^. The catalytic activity of PKA at the vicinity of CFTR is determined by the cAMP concentration, influenced by its production, degradation, and egress from the cells. These processes are defined by the activities of e.g. ACs, PDEs, and MRP2/4, associated with the CFTR macromolecular signalling complex^57–59^. There are nine mammalian genes (ADCY1-9) that encode membrane-bound AC isoforms and one soluble isoform (ADCY10). CFBE expresses both membrane-bound (AC1, 6, 7, and 9) and the soluble (AC10) AC isoforms according to our immunoblot analysis (Supplementary Fig. 2a).

**Figure 5:**
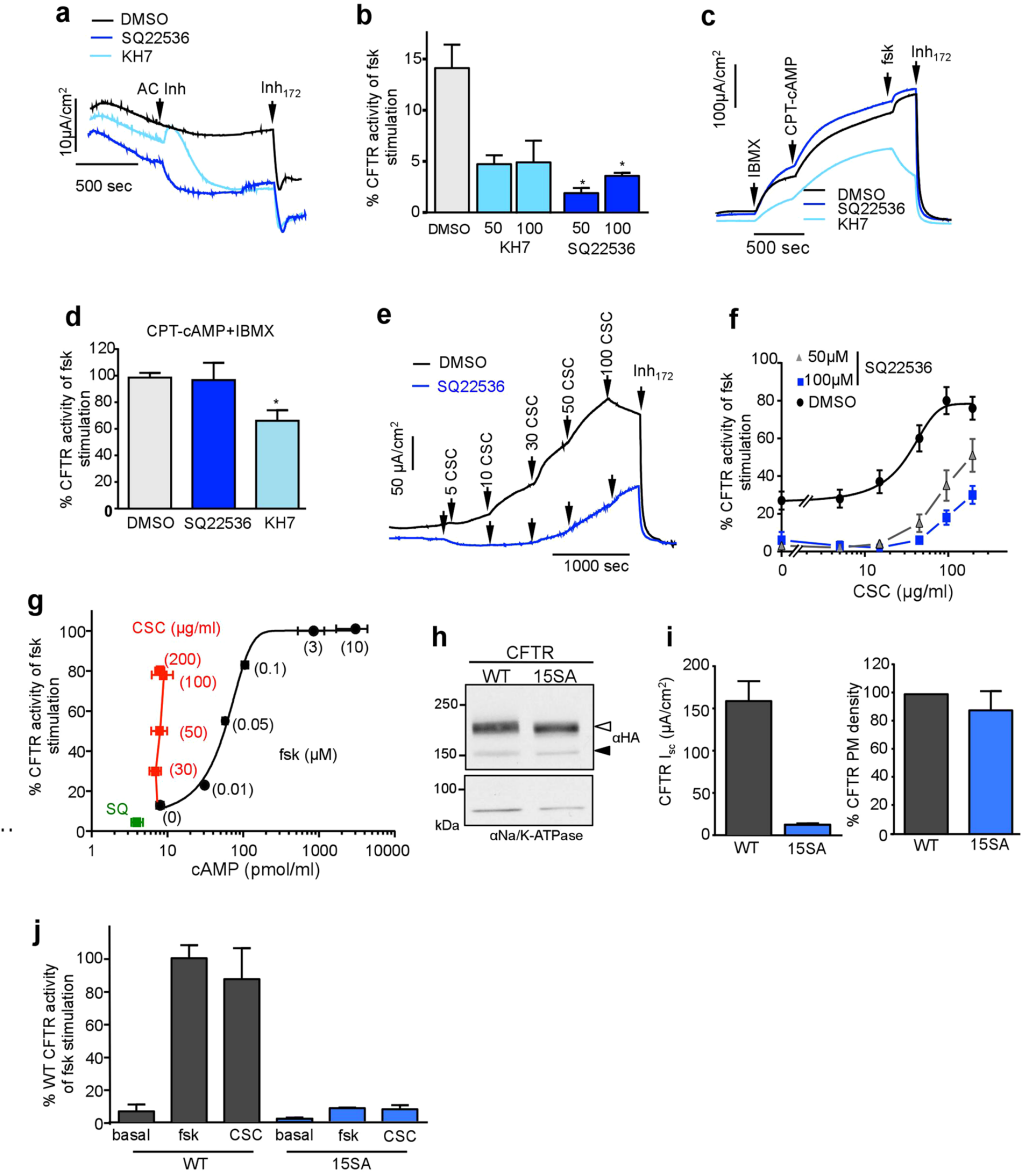
CFTR activation by CSC depends on adenylyl cyclase activity and PKA phosphorylation but does not increase intracellular cAMP. (**a**) Representative I_sc_ measurements depict the effect of 100 μM ACs inhibitors (KH7 and SQ22536) or DMSO on CFTR -mediated basal chloride current in WT-CFTR expressing CFBE. (**b**) Summary of ACs inhibitors (KH7 and SQ22536) effect on basal CFTR-mediated I_sc_ calculated as the percentage of maximal current reached after maximal forskolin stimulation compared to the baseline after blocking CFTR with Inh_172_. Data are means ± SEM, n = 3–16 *p < 0.05 (**c**) CFTR-mediated I_sc_ activation with the successive addition of phosphodiesterase inhibitor IBMX (200 μM), cell permeable cAMP agonist (CPT-cAMP; 500 μM) and forskolin (10 μM) on SQ22536 (100 μM), KH7 or DMSO pre-treated CFBE. (**d**) Summary of (**c**) Percentage of maximal current after CPT-cAMP and IBMX activation calculated as the percentage of maximal current reached after maximal forskolin stimulation compared to the baseline after Inh_172_. Data are means ± SEM, n = 3 *p < 0.05 (**e**) Representative I_sc_ current of CSC titration on DMSO (black) or SQ22536 (100 μM) pre-treated (blue) in CFBE. (**f**) Summary of SQ22536 (50 and 100 μM) pre-treatment on CSC-triggered CFTR activation in CFBE. Data are means ± SEM, n = 3 with technical duplicates (**g**) cAMP level and Inh_172_-sensitive I_sc_ in CFBE upon inhibition by SQ22536 (100 μM SQ, green) or stimulation by forskolin (0.01, 0.05, 0.1, 2 and 10 μM, black) or CSC (30, 50, 100 and 200 μg/ml red) measured with cAMP ELISA kit. Data are means ± SEM. n = 3(**h**) Immunoblot analysis of WT and 15SA PKA-phosphorylation deficient CFTR-3HA expression in CFBE. Complex- and core-glycosylated CFTR are indicated by an empty and filled arrowhead, respectively. Na/K ATPase was probed as a loading control. (**i**) 15SA mutations largely prevent the forskolin-induced CFTR activation. The WT and 15SA CFTR mediated I_sc_ (left panel) and PM density (right panel) were measured in CFBE. I_sc_ was measured in the presence of 20 μM forskolin. Data are means ± SEM, n = 3. Right panel: representative 15SA CFTR mediated I_sc_ upon CSC and forskolin stimulation. (**j**) Summary of WT- and 15SA-mediated I_sc_ current normalised for CFTR PM density and expressed as the percentage of forskolin-stimulated WT I_sc_ after 10 μM forskolin stimulation. Note that proportionally reduced activation of the 15SA CFTR was detectable by CSC (200 μg/ml) Data are means ± SEM, n = 4.

To probe the role of ACs in CFTR regulation by forskolin and CSC, we employed SQ22536 and KH7 blockers of transmembrane and soluble ACs, respectively^60^. Both KH7 and SQ22536 decreased the basal (or residual) CFTR channel activity from ~14% to ~2–5%, relative to the forskolin-stimulated I_sc_ in CFBE (Fig. 5a,b). To evaluate the non-specific effect of the inhibitors, PKA was directly activated by the cell-permeant CPT-cAMP in the presence of the phosphodiesterase inhibitor, IBMX. KH7 (100 μM), but not SQ22536 (100 μM) attenuated the activation of CFTR I_sc_ by CPT-cAMP + IBMX (Fig. 5c,d), suggesting that KH7 may interfere with CFTR activation non-specifically. Therefore, in subsequent experiments, we used the SQ22536.

Preincubation of CFBE with SQ22536 (50–100 μM, 40 min) reduced the CSC-activated CFTR I_sc_, consistent with the requirement of constitutive AC activity for the CSC-induced CFTR channel function (Fig. 5e,f). Next, we examined whether CSC-induced CFTR activation is accompanied by the global elevation of cytosolic cAMP concentration due to AC activation. Surprisingly, we were unable to detect significant alteration in the cytosolic cAMP concentration after CSC exposure (30–200 μg/ml, ~10 min) in CFBE using an enzyme immunoassay (EIA) (Fig. 5g and Supplementary Fig. 2b). This cannot be explained by the insensitivity of the EIA. We readily detected a ~3-fold increase in the resting cAMP level (from 8 ± 0.9 to 31 ± 10 pmol/ml, SEM, n = 4–10) even after 10 nM forskolin stimulation, which also led to a 12 ± 2% (SEM, n = 8) increase of CFTR I_sc_ relative to maximal forskolin activation (Fig. 5g). Furthermore, while maximally activating forskolin concentration has elicited >100-fold increase in resting cAMP level, inhibition of ACs by SQ22536 (45 min) decreased the resting cAMP level by 50% (from 8 ± 0.9 to 4 ± 1.2 pmol/ml, SEM, n = 5–10, Fig. 5g), concomitant with ~10% reduction of the constitutive CFTR I_sc_ (Fig. 5b). These results demonstrate that the cAMP EIA has the sensitivity to resolve small cAMP changes in the cytosol and raised the possibilities that the channel phosphorylation is achieved by CSC *via* localised PKA activation of the CFTR macromolecular signalling complex and/or by direct interaction, independently of PKA. Considering that monitoring of localized cAMP concentration by FRET detection of the Epac-cAMP sensor (CFP-Epac-YFP)^61^ was not feasible due to the high intrinsic fluorescence of the CSC, we used alternative approaches to evaluate the involvement of PKA activation in the downstream signalling of CSC.

We examined the impact of CSC on the 15SA-CFTR mutant variant, which is resistant to PKA activation^32^. While the 15SA-CFTR-3HA has a WT-like cellular and PM expression according to immunoblot analysis and PM-ELISA in CFBE (Fig. 5h,i), forskolin activated the 15SA-CFTR only to ~8% (13 ± 1.1 μA/cm^2^, n = 4) of the WT level (Fig. 5j). The comparable reduction in the CSC-induced I_sc_ (11 ± 1.6 μA/cm^2^, n = 4) of the 15SA-CFTR mutant, implies that phosphorylation of the PKA consensus sites is required for the CSE-elicited CFTR activation (Fig. 5i). Furthermore, we compared the CSC- and forskolin-stimulated phospho-occupancy of CFTR PKA consensus sites.

### Determination of CFTR phospho-occupancy in CFBE by mass spectrometry

**Figure 6:**
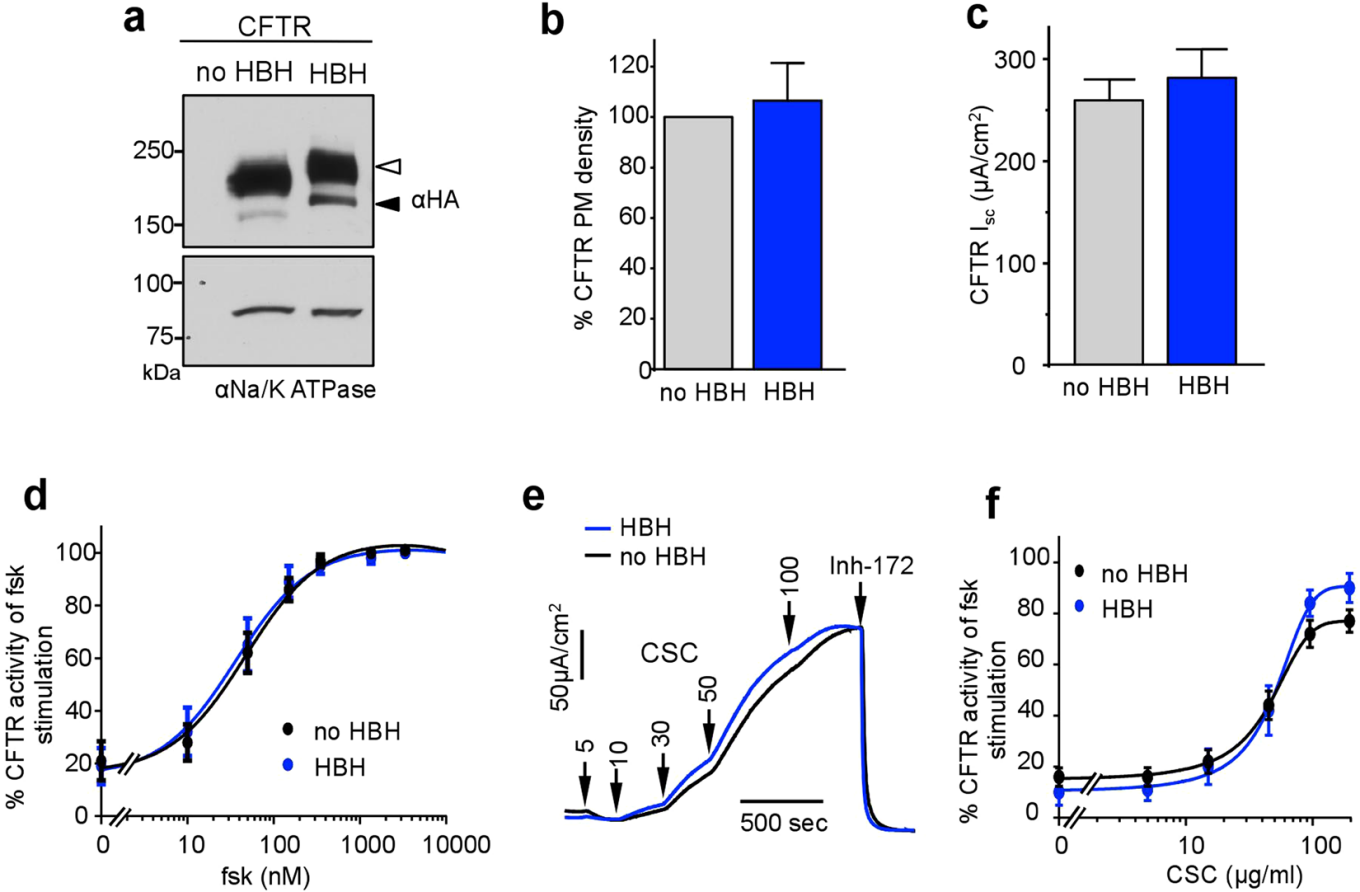
Characterisation of HBH-CFTR-3HA expression and function. (**a**) Immunoblot analysis of CFTR-3HA and HBH-CFTR-3HA CFTR expression in CFBE. Complex- and core-glycosylated CFTR are indicated by an empty and filled arrowhead, respectively. Na/K ATPase was probed as a loading control. (**b**) PM densities of CFTR-3HA and HBH-CFTR-3HA were measured in CFBE with cell surface ELISA assay ECL detection. Data are means ± SEM, n = 3. (**c**) The CFTR-3HA- and HBH-CFTR-3HA-mediated I_sc_ in the presence of 20 μM forskolin. (**d)** The summary figure of forskolin dose-response in WT-3HA and HBH-CFTR-3HA expressed in CFBE. Data are means ± SEM. n = 3 (**e**) Representative I_sc_ recordings in WT-3HA- or HBH-CFTR-3HA- expressing CFBE. Arrows represent sequential addition of 5, 10, 30, 50, and 100 μg/ml CSC followed by addition of 20 μM Inh_172_. (**f)** Summary of (**e**) Dose response curve of CSC on Inh_172_-sensitive I_sc_ in CFBE. Data represent means ± SEM, n = 3.

To conclusively demonstrate that CSC exposure leads to PKA-dependent phosphorylation of CFTR, we implemented a novel CFTR affinity enrichment method, followed by an advanced LC-coupled tandem mass spectrometry (MS) technique that uses EThcD-based fragmentation. To this end, we genetically engineered the His_6_-BIO-His_6_ (HBH) tag^62^ at the N-terminus of CFTR (HBH-CFTR-3HA) and expressed the channel in CFBE. We verified that the HBH-CFTR-3HA preserves the biochemical and functional characteristics of the CFTR-3HA, determined by immunoblotting (Fig. 6a), PM-ELISA (Fig. 6b), and Ussing measurements (Fig. 6c), respectively. Monitoring the I_sc_ activation showed that the forskolin (Fig. 6d) and CSC (Fig. 6e,f) dose-response curves of HBH-CFTR-3HA were comparable to that of the CFTR-3HA.

To analyse the phospho-occupancy, HBH-CFTR-3HA was affinity-purified on streptavidin bead from control and forskolin-treated CFBE. After the label-free MS data acquisition, we performed a targeted, relative quantification of the phospho-occupancy, which involved quantifying the ratio of all phosphorylated and non-phosphorylated peptides that contained PKA consensus phosphosites. To ensure the confidence of each phosphosite localisation (S422, S660, S670, S686, S700, S712, S737, S753, S768, and S795), we manually verified the MS/MS fragmentation spectra for either +80 Da or −98 Da mass shifts (Supplementary Fig. 3). We identified ten previously validated PKA sites (Supplementary Table 1).

**Figure 7:**
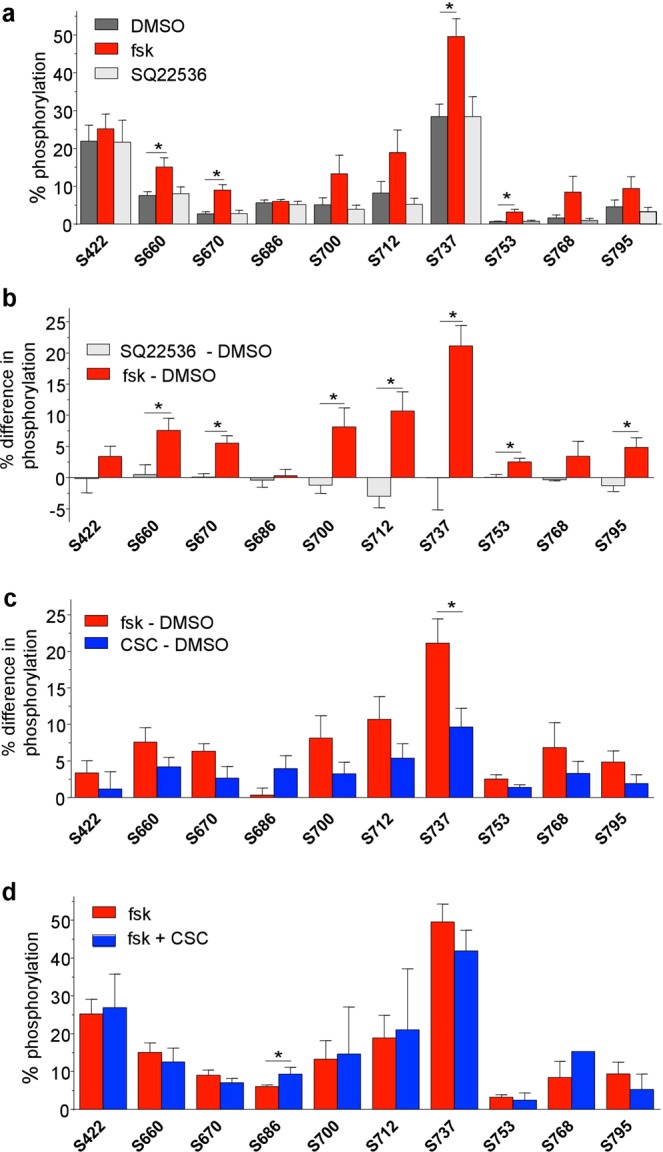
Phospho-occupancy of identified PKA sites in CFTR under various conditions. (**a**) Relative phosphorylation (%) or phospho-occupancy in the vehicle (DMSO-treated cells), forskolin (10 μM, 10 min), and SQ (50 μM, 40 min) treated cells. The phospho-occupancy of each site was calculated as described in the methodology section. (**b**) Differential phosphorylation levels or Delta phosphorylation (Δ%) between the AC-stimulated (forskolin, red) and AC-inhibited (SQ22536, grey) conditions, corrected for the basal phosphorylation values (DMSO). (**c**) Differential phosphorylation levels or Delta phosphorylation (Δ%) between the forskolin and CSC-treated (200 μg/ml, 20 min) samples corrected for the basal phosphorylation values. (**d**) Relative phosphorylation (%) of CFTR purified from forskolin- and forskolin + CSC-treated CFBE. Each bar plot in all the panels represents the mean ± SEM of at least three biological replicates. Unpaired t-tests were performed in GraphPad Prism 6.0 on each phosphosite between two different treatment conditions. P-values of <0.05% were considered significant.

We quantified the relative phospho-occupancy in CFTR-derived peptides from DMSO and forskolin or SQ22536 treated CFBE by calculating the percent phosphorylation for each of the ten individual sites as described in Methods. Firstly, we analysed the effect of forskolin (10 μM, 10 min, 37 °C) on CFTR phospho-occupancy. Most of the sites, except S422, S686 and S753 displayed ~5–20% elevated phosphorylation levels upon forskolin stimulation (Fig. 7a,b), consistent with the model that phosphorylation of multiple sites synergistically contributes to the maximal channel activation^63,64^.

Intriguingly, the high constitutive phospho-occupancy of S422 and S737 (~20–30%) was not decreased by inhibiting the AC activity with SQ22536 (50–100 μM, 20–40 min) and only ~1–3% reduction was observed for four additional sites (S686, S700, S712, and S795) (Fig. 7b). Whether these changes alone are enough to explain the observed attenuation of the constitutive I_sc_ under identical conditions will require further investigation. We also compared the phosphorylation levels between forskolin- and SQ22536-treated samples following correction with the phosphorylation levels in the presence of DMSO (Fig. 7b). Seven consensus sites (S660, S670, S700, S712, S737, S753 and S795) showed a significant increase in their phosphorylation level upon activation of the adenylyl cyclase/cAMP/PKA signalling pathway (Fig. 7b).

### CFTR phospho-occupancy upon CSC-induced activation and inhibition

Having established the phosphorylation levels in the basal and forskolin-stimulated states of CFTR, we compared the CSC (200 μg/ml, 25 min) and forskolin effect on CFTR phospho-occupancy, after correction for the constitutive phospho-occupancy. Although eight of the PKA sites showed a tendency to display lower phospho-occupancy upon CSC treatment in comparison to forskolin exposure, the differences were not significant (Fig. 7c). Only S737 showed a significant difference between CSC- and forskolin-mediated stimulation, with a ~10–15% decreased phospho-occupancy upon CSC treatment (Fig. 7c). Jointly, these results strongly suggest that acute CSC can elicit compartmentalised PKA activation and a phosphorylation pattern of the CFTR similar to that rendered by forskolin stimulation in its macromolecular signalling complex in the absence of global elevation of the cytosolic cAMP (Fig. 5g).

CFTR phospho-occupancy was also measured after maximal inhibition of the forskolin-stimulated channel I_sc_ by CSC (Fig. 7d). No significant reduction in CFTR phospho-occupancy was detected. This suggests that channel dephosphorylation does not account for the CSC-induced acute I_sc_ inhibition.

### CSC inhibits MRP4 transport function

The plasma membrane MRP4 transporter, a member of the CFTR macromolecular signalling complex, can extrude cAMP among other substrates from the cytosol^29–31^. Suppressing MRP4 activity by the specific inhibitor, MK571, resulted in localised intracellular elevation cAMP and augmented CFTR activation^65^. Considering the observed phospho-occupancy elevation of CFTR without detectable cytosolic cAMP changes, we presumed that CSC might hinder the extracellular transport of cAMP *via* the MRP4 and results in localised PKA activation. This consideration seems plausible, considering that CSC-induced acute activation of CFTR was suppressed by the PKA inhibitor H89 (Fig. 1c). Furthermore, the MRP4 transcript levels in CFBE, NCI-H441, and CR-HBE cells were similar to that established for the CaCo-2 epithelial cells by qPCR analysis (Supplementary Fig. 5)^66,67^.

**Figure 8:**
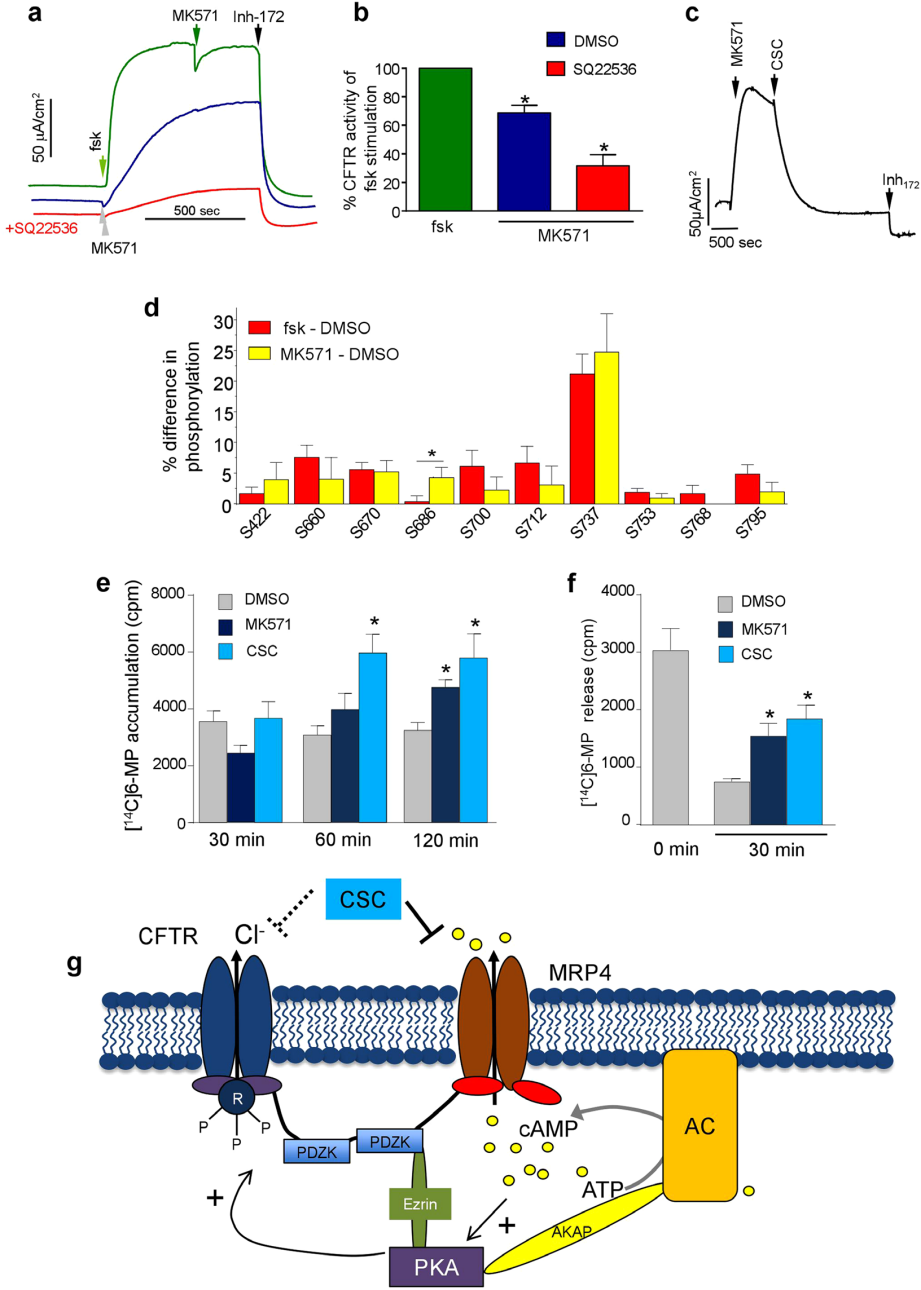
CSC activates CFTR in a compartmentalised fashion *via* inhibiting the cAMP egress through MRP4. (**a)** Addition of forskolin (green) or the MRP4 inhibitor, MK571 (50 μM, blue), activates CFTR dependent I_sc_. Pre-treatment of adenylyl cyclase inhibitor SQ22536 (100 μM, red) decreased the MK571 caused activation. (**b**) Summary of MK571 activated CFTR-mediated current with and without SQ22536 pre-treatment compared to forskolin stimulation. (**c**) Representative I_sc_ trace in CFBE shows the effect of CSC on MK571 stimulated current. Addition of MK571 followed by CSC (200 μg/ml) and Inh_172_ (20 μM). (**d**) Differential phosphorylation levels or Delta phosphorylation (Δ%) between the forskolin and MK571-treated samples corrected for the basal phosphorylation values. Bars represent the mean ± SEM of at least three biological replicates. (**e**) Time-dependent accumulation of MRP4 substrate, [^14^C]-6-meracptopurine (10 μM, [^14^C]6-MP) in CFBE after 30, 60, and 120 min in the presence of DMSO, MK571 (50 μM) or CSC (200 μg/ml) respectively. Cell-associated radioactivity was counted after treatments. *p < 0.05 vs DMSO (**f)** Efflux of [^14^C]6-MP from CFBE. Cells were first incubated for 45 min in the presence of 10 μM [^14^C]6-MP. The medium was then changed to [^14^C]6-MP free medium with DMSO/50 μM MK571/200 μg/ml CSC and cell-associated [^14^C]6-MP was measured after 30 min *p < 0.05 vs DMSO. (**g**) Schematic representation of the CSC interaction with the CFTR macromolecular signalling complex at the apical plasma membrane of airway epithelia. Abbreviations: CSC, cigarette smoke condensate; MRP4, multidrug resistance-associated protein 4; PDZK, PDZ kinase; AKAP, A-kinase-anchoring protein; AC, adenylyl cyclase; ATP, adenosine triphosphate; cAMP, 3′,5′-cyclic adenosine monophosphate.

To test the physiological relevance of MRP4 cAMP translocation, CFBE were incubated with the potent MRP4 blocker, MK571^68^. MK571 rapidly activated the CFTR I_sc_, which was significantly attenuated by the inhibition of the ACs by SQ22536 (Fig. 8a,b). Furthermore, the MK571-activated CFTR I_sc_ was highly susceptible to CSC inhibition (Fig. 8c), similar to the forskolin activated channel (Fig. 2c), consistent with the PKA-mediated phosphorylation of CFTR under this condition. This inference was confirmed by comparing CFTR phospho-occupancy after MK571 or forskolin treatment by MS. We found that only S686 of the ten CFTR consensus PKA phosphosites, displayed significantly different phosphorylation in MK571 -treated relative to that in forskolin-exposed CFBE (Fig. 8d). This observation supports the notion that MRP4 inhibition by CSC may induce the subcompartmentalized PKA catalysed phosphorylation of CFTR.

Finally, we demonstrated that CSC could indeed interfere with the MRP4-mediated cAMP egress from CFBE. We monitored the intracellular accumulation of the radiolabelled 6-Mercaptopurine ([^14^C]6-MP*)*, an established substrate of the MRP4 transporter^31,69^. Accumulation of [^14^C]6-MP was markedly enhanced in the presence of 200 μg/ml CSC, as in the presence of MK571 (Fig. 8e). The impeded cellular efflux of [^14^C]6-MP *via* the MRP4 accounts for the substrate intracellular accumulation, an inference confirmed by directly monitoring the cellular loss [^14^C]6-MP from CFBE in the presence or absence of CSC or MK571 (Fig. 8f). The efflux of [^14^C]6-MP from pre-loaded CFBE was inhibited by 62% and 45% with MK571 and CSC, respectively (Fig. 8f). These observations provide support for the hypothesis that localised accumulation of cAMP is, at least partly, achieved by the inhibition of MRP4 activity with CSC.

## Discussion

While subacute and chronic CS and CSC exposure is associated with impaired CFTR function and constitute major risk factors for COPD, CF, and other lung diseases^8,70^, our knowledge of the short-term CSC impact on the ion transport of respiratory epithelia, a critical determinant of the ASL homeostasis and mucociliary transport (MCT), is incomplete. Here, we uncover the mechanism of the robust upregulation of anion secretion upon acute CSC exposure of CFBE and primary CR-HBE, which was critically dependent upon the expression of functional CFTR. The activation of the resting CFTR by CSC exposure offers a plausible explanation for the accelerated MCT of primary HBE upon incubation with organically soluble CS extract^53^. The activation, however, after 24 h incubation with CS or CS extract exposure was followed by the MCT inhibition, which was associated with transcriptional and posttranslational downregulation of CFTR^53^, in line with other reports^11,17,52,53^. Considering the compositional^34^ and epithelial signalling differences in the CSC and CSE^35^ effect, it is plausible that constituents of both CSE and CSC contribute to the transient activation of the MCT upon CS exposure^53^.

There are notable differences in CFTR activation pattern by CSC or CSE. While comparably fast activation and relatively slower inactivation kinetics of I_sc_ was observed by CSC and CSE stimulation of CFBE, primary HBE displayed a significantly attenuated magnitude and duration of CFTR I_sc_ activated by CSE^35^ as compared to that of CSC in our study. This may be explained by the differences in the mechanism of PKA activation. The CSE-induced ROS generation and Ca^2+^ release from the ER represent signalling pathways that are transient in nature, while the MRP4 inhibition by CSC appears to be longer lasting under our experimental condition and could not be reversed by extensive washings. Our findings with the following observations suggest a complex modulatory mechanism for CFTR activation in concert with MRP4 inhbition, representing a first line defence against inhaled toxic agents and oxidants of CS in airway epithelia, which is eventually thwarted by the subacute inhibition of CFTR, as well as by the pleiotropic long-term effects (increased in endoplasmic reticulum stress, unfolded protein response, mucus secretion, apoptosis, as well as impaired MCT and CFTR downregulation) of CS exposure^8,71^.

Acute exposure of CSC leads to PKA-dependent channel activation of the non-phosphorylated CFTR to 40–80% of the supramaximal forskolin-stimulated CFTR I_sc_ in immortalised and primary HBE. Consistent with the causal role of PKA-mediated phosphorylation, H89, a specific PKA inhibitor, as well as mutagenesis of fifteen consensus PKA phosphorylation sites (CFTR-15SA), reduced both the forskolin and the CSC-induced I_sc_ activation by ~85% without altering CFTR PM expression level. Surprisingly, CFTR activation by CSC prevailed in the absence of detectable elevation of the cytosolic cAMP concentration, *nota bene* the dynamic range of the cAMP-EIA spanned from ~4 to ~3000 pmol/ml cAMP concentration and resolved changes in cAMP cellular level that was enough to elicit a ~10% increase in CFTR I_sc_ relative to forskolin activation. CFTR activation by CSC was retained upon exposing the cells to reducing agents and persisted in the absence of cytosolic cAMP elevation. Based on these observations and with the established subcompartmentalised phosphoregulation of the CFTR macromolecular signalling complex, consisting of ACs, phosphodiesterases, and MRP4 transporter that mediates cAMP egress from the cytoplasm^72^, we propose that inhibition of MRP4 by CSC, at least partly, accounts for the localised PKA activation of CFTR. In contrast, CSE initiates prostanoid synthesis by reactive oxygen species (ROS), generated by NADPH oxidase, leading to global, though limited increase in the cytosolic cAMP concentration *via* autocrine activation of EP4 prostanoid receptors and store-operated Ca^2+^-signalling^35^.

The MRP4 transporter is tethered to the CFTR macromolecular signalling complex *via* PDZK1, a PDZ domain protein^72,73^. Thus, the elevation of the cAMP level is the result of inhibition of cAMP efflux *via* MRP4 transporter in concert with the constitutive AC activity in association with the CFTR macromolecular complex. In support, both MRP4 blocker (MK571), as well as the CSC accelerated the 6-MP uptake into CFBE and inhibited its extrusion from 6-MP pre-loaded CFBE. Last, but not the least, the phospho-occupancy pattern of CFTR was similar in the presence of CSC or the MRP blocker (MK571) to that of forskolin stimulation. A similar subcompartmentalised CFTR activation has been proposed for an anti-colon cancer drug (irinotecan) and an anti-retroviral drug (3′-azido-3′-deoxythymidine) using intestinal fluid secretion in closed-loop diarrhoea model and enterospheres, isolated from wild-type (*Mrp4*^+/+^) and *Mrp4*^−/−^ mice^72^. These drugs not only inhibited the transport activity of MRP4, but also augmented the physical interaction between MRP4 and CFTR *via* PDZK1, therefore, further restricting the localised increase of the cytosolic cAMP concentration^72^, which may also prevail after CSC exposure. Our results, however, do not formally preclude the contribution of inhibition of other cAMP exporters (ABCC5 and ABCC11)^74^ and phosphodiesterases to CFTR activation. Since these transporters are not recognized constituents of the CFTR macromolecular signalling complex, their transport inhibition by CSC is expected to be associated with global cAMP elevation.

To compare the CFTR phospho-occupancy upon CSC and forskolin exposure, it was necessary to implement an effective CFTR enrichment method that relies on the HBH affinity-tag in concert with an EThcD-based tandem MS technique. Using this targeted quantification approach, we determined the percentage of changes in the phospho-occupancy of PKA consensus sites relative to the respective total phosphosite-containing peptide count, identified from affinity-purified CFTR. Here we document the phospho-occupancy of ten phosphosites (S422 in NBD1 and S660, S670, S686, S700, S712, S737, S753, S768, S795 in the RD) in CFTR isolated from CFBE (Supplementary Table 1). We were unable to reliably monitor the phospho-occupancy of the remaining five PKA phosphorylation sites (T690, T787, T788, S790, and S813) amongst the 15 PKA consensus sites, either due to low MS/MS signal quality or lack of tryptic peptide coverage. Nevertheless, this is a considerable improvement in comparison to the collision-induced dissociation (CID)-based SRM technique that reported peptides carrying ten PKA consensus sites in purified CFTR, but only two phosphorylation sites were detectable (S660 and S737) in both the resting and stimulated state in BHK cells^33^ (Supplementary Table 1) and partly attributed to the use of EThcD, a dual fragmentation technique, which provides enhanced peptide ion fragmentation and identification.

The mechanism of MRP4 inhibition remains to be established. Besides covalent modification of MRP4 by reactive compounds in the CSC, it is possible that other member of the multidrug resistance ABC transporter subfamily (e.g. MRP1/2) may modulate CFTR activation. MRP1/2 can translocate a multitude of substrates, including tobacco-specific carcinogens (TSCs) and/or other CS constituents that may compete with cAMP for translocation^75^. Furthermore, it is also plausible that activation of MRP2 upon CSC accumulation, including the nicotine-derived 4-(methylnitrosamino)-1-(3-pyridyl)-1-butanone (NNK) transport substrate, may contribute to CFTR activation by PKA-mediated phosphorylation *via* the functional interaction of MRP2-CFTR, relying on PDZ-domain proteins, as observed in lung epithelia^75^.

Considering the acquired loss-of-function CFTR phenotype of HBE in COPD and chronic bronchitis, understanding the molecular basis of acute and extended CS exposure on CFTR inhibition has important therapeutic implications. The first indication that CS can acutely inhibit Cl^−^ secretion in airway was observed on dog trachea in 1983^15^. Three hallmarks of the inhibition were recognised: (1) The inhibition by CS was more pronounced after tracheal stimulation by epinephrine. Consistently, we found that CFTR activation with forskolin accelerated the onset and extent of the CSC inhibitory effect in CFBE. (2–3) Antioxidants and oxygen radical scavengers were ineffective to protect against acute inhibition of Cl^−^ secretion, which observation with the reversibility of the I_sc_ suppression suggest that oxidative modification of the channel likely does not account for the acute inhibition by CSC. In line, we were unable to detect posttranslational modifications (Schiff’s base modification and a Michael addition on lysines) of CFTR, isolated from CSC exposed CFBE (data not shown). The possibility that increased phosphatase activity of the PP2A was responsible the channel inactivation by CSC, as reported after CS or CSE exposure of mice lung or human small airway epithelia, respectively^76^, is unlikely because of the preserved phospho-occupancy of the inhibited channel, measured by MS following forskolin plus CSC treatment of CFBE. Since the inhibitory effect is reversed in the absence of CSC, it is more likely that the effect is caused by component(s) of CSC itself. By the same token, one can also rule out impeded phosphorylation, which is consistent with the observed CFTR inhibition with CSC in phospholipid bilayers, containing negligible phosphatase activity^51^. The documented reversible channel inhibition by acute CSC exposure is consistent with the observations that the gating potentiators, VX-770, can partially rescues CFTR activity after 24 h of CS extract exposure in primary HBE^77^. The progressive long-term functional and biochemical downregulation of CFTR at the transcriptional and posttranslational level by chronic CS treatment, however, will require the implementation of additional or alternative therapeutic approaches to alleviate the compromised channel activity at the apical PM of airway epithelia.

## Methodology

### Antibodies and reagents

N-acetyl cysteine and ATP were purchased from Sigma, Glutathione monoethyl ester from Calbiochem, KH7 and SQ22536, forskolin, Inhibitor-172, MK571 and irinotecan were purchased from Tocris. [^14^C] 6-Mercaptopurine (6-MP) (51 mCi/mmol) was derived from Moravek Biochemicals, CA. ADCY1, 3,6,7,9 and10 adenylyl cyclase antibodies were purchased from Abcam.

### Cell culture

CFBE41o- (CFBE) bronchial epithelial cell line was a kind gift from Dr. Gruenert and were propagated in MEM medium (Gibco) supplemented with 10% FBS, 5 mM L-Glutamine and 10 mM HEPES (Invitrogen) on fibronectin-coated plastic dishes as described^38^ For experiments, cells were seeded and differentiated for at least four days on coated plastic wells or polyester-permeable supports (Transwell and Snapwell filters Corning). NCI-H441, human papillary adenocarcinoma epithelial cells (ATCC HTB-174^TM^), were cultured in DMEM (Invitrogen) supplemented with 10% FBS and 5 mM sodium pyruvate.

Human bronchial epithelial (HBE) cells were isolated from bronchi according to protocols approved by the Human Research Protection Program Institutional Review Board of UCSF (No 10-02253) or were purchased from the Cystic Fibrosis Translational Research centre (CFTRc), McGill University. Following the previously published protocol^41^, HBE cells were conditionally reprogrammed. Briefly, the HBE cells were cultured on irradiated 3T3-J2 fibroblast in F-medium supplemented with 10 µM ROCK-inhibitor, Y-27632. After the expansion, cells were seeded on collagen IV-coated snapwell filter supports (Corning) and differentiated at air-liquid interface in Ultroser G™ medium for over four weeks with basolateral medium change every 2–3 days^42^. All cell lines were maintained in a 37 °C CO_2_ incubator. Cell lines expressing inducible WT-CFTR with a 3HA tag (CFTR-3HA) were generated using the ClonTech pLVX-Tight-Puro lentivirus technology, as described previously and the CFTR expression was induced with 50–250 ng/ml doxycycline for at least 4days.

### Cigarette smoke extracts

Cigarette smoke condensate (CSC) was purchased from Murty Pharmaceuticals (Lexington, USA). According to the manufacturer’s description, CSC was prepared by smoking University of Kentucky’s 3R4F Standard Research Cigarettes on an FTC Smoke Machine through a Cambridge filter. Cambridge filters can retain 99% of particulate matter larger than 0.1 μm that have been emitted during the combustion of the cigarette. The Total Particulate Matter (TPM) on the filter is calculated by measuring its weight, and the condensate was extracted by soaking and sonication in DMSO to reach a 40 mg/ml concentration in solution.

### Cytosolic cAMP measurement

Intracellular cAMP concentrations were evaluated with cAMP-enzyme immunoassay (EIA) kit (ENZO Life Sciences) following the manufacturer instructions. Confluent CFBE monolayers were grown in 6-well dishes for at least 4 days, treated (SQ22536 50 μM, 45 min; forskolin-20 μM, 10 min; CSC-200 μg/ml, 20 min), and washed with ice-cold PBS supplemented with 1 mM MgCl_2_ and 0.1 mM CaCl_2_ (PBS++) and then lysed with 350 µl lysis medium (0.1 M HCl −0.5% Triton-X). 100 µl of samples or known concentration standards were added to wells coated with GxR-IgG affinity-purified antibody. cAMP-conjugated with alkaline phosphatase is then added, followed by a rabbit antibody to cAMP. After 2 h incubation, the antibody binds competitively to the cAMP in the sample or to the conjugate. The plate was then washed leaving only the bound cAMP. The yellow-coloured substrate formed on the conjugate when catalysed by alkaline phosphatase. The more yellow the sample, the less cAMP is present in it. The colourimetric reaction was detected at 405 nm by spectrophotometer (TECAN). Quantification of the cAMP level was done against a calibration curve that was plotted using the cAMP standards, included in the kit. All experiments are biological triplicates, including two technical repeats in each experiment.

### Cell surface CFTR quantification

CFTR PM density was determined by cell surface ELISA and biotinylation techniques as descreibed previously and detailed in the Supplementary Methods.

### Short circuit current measurement

Short circuit current measurements (I_sc_) were performed on CFBE, NCI-H441, and CR-HBE as described in our previous publications^41^ and in the Supplementary Methods. The CFBE monolayers integrity were maintained during the I_sc_ measurements, as indicated by the baseline, forskolin and Inh_172_-treated TEER values in the absence (588 ± 125, 188 ± 26 and 763 ± 128) or presence of CSC (450 ± 66, 790 ± 91), respectively.

### Sample purification for tandem mass spectrometry analysis

HBH-CFTR-3HA-expressing CFBE monolayers were grown in fibronectin-coated 6-cm dishes at least four days post-confluency. CFTR expression was induced with doxycycline (250 ng/ml) for four days. After treatments (SQ22536-100 µM, 20–40 min; forskolin-10µM, 10 min; CSC-200 µg/ml,20 min; MK571-50 µM,10 min in KRB at 37 °C), cells were placed on ice and washed with ice-cold PBS++ supplemented with phosphatase inhibitor cocktail (2 mM Sodium fluoride, 2 mM imidazole, 1.15 mM Sodium-molybdate, 4 mM Sodium orthovanadate, 4 mM Sodium tartrate, 1 mM Sodium pyrophosphate, 1 mM β-Glycerophosphate) for 1–2 min. Cells were then lysed with lysis buffer (0.4% Triton X-100, 300 mM NaCl; 20 mM Tris pH 8.0 + 1 mM DTT) supplemented with protease (10 µM Leupeptin, 10 µM Pepstatin A, 100 µM PMSF), kinase inhibitors (4 mM EDTA and 4 mM EGTA) and phosphatase inhibitor cocktail for 5 min. Lysates were collected in pre-chilled Eppendorf tubes centrifuged at 14000 rpm for 10 min, and the supernatant was then allowed to bind to Dynabeads® MyOne™ Streptavidin C1 (Thermo Fischer Scientific) for an hour with end-to-end rotation at 4 °C. To remove as much non-specifically bound proteins, the beads were washed as follows: 1x with lysis buffer, 1x high salt buffer (0.4% Triton X-100, 500 mM NaCl; 20 mM Tris pH 8.0, 1 mM DTT) 1x with 6 M Urea (0.4% Triton X-100, 300 mM NaCl, 20 mM Tris pH 8.0, 6 M Urea, 1 mM DTT), 2x with Dodecyldimethylglycin (DMNG)-containing solution (300 mM NaCl, 20 mM Tris pH 8.0, 0.01% DMNG) to eliminate Triton X-100 and 3x with 50 mM ammonium bicarbonate supplemented with 0.01% DMNG. After washing, the bead-bound protein samples were kept on ice in 50 mM ammonium bicarbonate until digestion.

### On-bead digestion and LC-MS/MS

The on-bead proteins were first diluted in 2 M Urea/50 mM ammonium bicarbonate, and on-bead trypsin digestion was performed overnight at 37 °C. The samples were then reduced with 13 mM dithiothreitol at 37 °C and, after cooling for 10 min, alkylated with 23 mM iodoacetamide at room temperature for 20 min in the dark. The supernatants were acidified with trifluoroacetic acid and cleaned from residual detergents and reagents with MCX cartridges (Waters Oasis MCX 96-well Elution Plate) following the manufacturer’s instructions. After elution in 10% ammonium hydroxide /90% methanol (v/v), samples were dried with a Speed-vac, reconstituted under agitation for 15 min in 12 µL of 2%ACN-1%FA and loaded into a 75 μm i.d. × 150 mm, Self-Pack C18 column, installed in the Easy-nLC II system (Proxeon Biosystems). Peptides were eluted with a two-slope gradient at a flow rate of 250 nl/min. Solvent B first increased from 1 to 36% in 66 min and then from 36 to 90% B in 14 min. The HPLC system was coupled to Orbitrap Fusion mass spectrometer (Thermo Scientific) through a Nanospray Flex Ion Source. Nanospray and S-lens voltages were set to 1.3–1.8 kV and 50 V, respectively. Capillary temperature was set to 225 °C. Full scan MS survey spectra (m/z 360–1560) in profile mode were acquired in the Orbitrap with a resolution of 120,000 with a target value at 1e6. The most intense peptide ions were fragmented by both HCD and EThcD and analysed in the linear ion trap with a target value at 2e4, and normalized collision energy at 28 V. An MS3 scanning was performed upon detection of a neutral loss of phosphoric acid (48.99, 32.66 or 24.5 Th) in HCD MS2 scans. The duty cycle was set to 3 seconds, and target ions selected for fragmentation were dynamically excluded for 30 sec after 3 MS/MS events.

### CFTR peptide identification and phosphorylation quantification

The peak list files were generated with Proteome Discoverer (version 2.1) using the following parameters: minimum mass set to 500 Da, maximum mass set to 6000 Da, no grouping of MS/MS spectra, precursor charge set to auto, and the minimum number of fragment ions set to 5. Protein database searching was performed with Mascot 2.6 (Matrix Science) against the UniProt human protein database as well as a user-defined CFTR database (April 15th, 2015). The mass tolerances for precursor and fragment ions were set to 10 ppm and 0.6 Da, respectively. Trypsin was used as the enzyme allowing for up to 1 missed cleavage. Cysteine carbamidomethylation was specified as a fixed modification and methionine oxidation as variable modifications. Data interpretation was performed using Scaffold (version 4.8).

Phosphorylated CFTR peptides and their non-phosphorylated counterparts were quantified using Pinnacle software (Optys Technologies). The protein/peptide results from Mascot (*.dat files) were combined with the associated raw data files (*.raw files) from the mass spectrometer using Pinnacle. Pinnacle automatically extracts ion chromatograms (XIC’s) for each detected peptide and integrates the area under each XIC in counts. Counts were then normalised for total ion current. The file areas of each peptide were normalised between treatments to the total CFTR reads in the respective treatment group. The phospho-occupancy or the percent of relative phosphorylation of each site was calculated as a ratio of all phosphorylated and unphosphorylated peptides that contained a given phosphosite, i.e., % phosphorylation of site A = [area of peptides phosphorylated at site A /sum of areas of all peptides carrying site A].

### Phosphorylation site localization

Scaffold PTM (Proteome Software, Portland, Oregon, USA) was used to annotate phosphorylation sites derived from MS/MS sequencing results obtained using Scaffold (version Scaffold_4.6.2). Using the site localisation algorithm^78^, Scaffold PTM re-analyses MS/MS spectra identified as modified peptides and calculates the A-score values and site localisation probabilities to assess the confidence of PTM site localisation. Scaffold PTM then combines localisation probabilities for all peptides containing each identified PTM site to obtain the best-estimated probability that a PTM is present at that site. In addition to the A-score values, the MS/MS spectra of each phosphosite were also inspected manually.

### MRP4 functional assay

Drug accumulation and efflux experiments were performed by a modified method as described^79^. Briefly, for accumulation experiments, CFBE cells were seeded in triplicates in 24-well plates for at least four days post-confluency in the presence of doxycycline (250 ng/ml). Cells were pre-treated for 10 min with DMSO, MK571, or CSC, and then incubated at 37 °C with [^14^C]6-MP (10 μM) in a complete medium for 1–2 h in the presence of DMSO, MK571 (50 μM) or CSC (200 μg/ml), respectively. Cells were then washed three times on ice with ice-cold PBS++ and lysed with 200 μl RIPA buffer for 5 min. Cell lysates were collected into a scintillation cuvette. Radioactivity was then measured by liquid scintillation counting. For efflux experiments, CFBE cells were seeded in triplicates in 24-well plates and were incubated first for 45 min with [^14^C]6-MP (10 μM) in a complete medium at 37 °C. The cells were then washed with full medium three times and were incubated at 37 °C for 30 min without [^14^C]6-MP. Cells were washed on ice with ice-cold PBS++, lysed, and then the intracellular radioactivity was counted from cell lysate.

### Quantitative RT-PCR

Total RNA was extracted from polarised CFBE cells grown on plastic in 12-well plates using miRNeasy Mini Kit (Qiagen, Hilden, Germany) and analysed using the one-step QuantiFast SYBR Green RT-PCR Kit (Qiagen) following the manufacturer’s recommendations. Data were analysed by efficiency-corrected comparative quantification with MxPro QPCR software (Agilent, Santa Clara, California, USA). MRP4 (ABCC4) was detected with primers sense ATTATTGATGAAGCGACGGC and antisense GCAAAACATACGGCTCATCA. Variations in RNA loading amount were accounted for by normalising to GAPDH (sense primer, CATGAGAAGTATGACAACAGCCT; antisense primer, AGTCCTTCCACGATACCAAAGT).

### Planar lipid bilayer studies

Planar lipid bilayers were formed, and CFTR-containing microsomes (containing 10–20 μg total protein) were fused to bilayers as described previously^80^.

### Cellular redox state measurements

To measure the GSH redox potential in CFBE, cells with the inducible expression of WT-CFTR were transduced with lentiviral particles encoding the cytosolic ratiometric redox sensor, glutaredoxin 1-redox-sensitive GFP (Grx1-roGFP) fusion protein^49^ to measure the GSH redox potential. Grx1-roGFP was inserted into pLVX-IRES-Hyg lentivirus expression vector using NotI/XbaI restriction sites. Grx1-roGFP and CFTR expressing CFBE cells were grown in 96-well plates and kept for at least four days post-confluency. Fluorescence intensities were measured at 520 nm (±20 nm) emission and at 405 nm and 488 nm excitation wavelengths, using a TECAN Infinite M1000 fluorescence plate reader at room temperature. A higher 405/488-ratio value represents a higher oxidative state. The redox probe maximum and minimum emission ratios were determined by addition of H_2_O_2_ (100 µm) and DTT (500 µM), respectively, as references. We were unable to determine the acute CSC effect on the intracellular redox state using the Grx1-roGFP sensor. This was due to the acquired autofluorescence of cells after CSC exposure, which masked the specific detection of Grx1-roGFP fluorescence even after eight washes of the CSC-exposed cells.

### Statistical analysis

Data are shown as mean ± SEM of n observations. GraphPad Prism 6 software (GraphPad Software Inc.) was used, and unless otherwise specified, for preparing the graphs and statistical analyses. Two-tailed p-values were calculated at 95% confidence level using the student t-test. Unpaired t-tests were used to compute the significance of differences in the phospho-occupancies between two treatment conditions. p-values < 0.05 were considered significant.

## Supplementary information

SUPPLEMENTARY INFO
