# Supplementary material for: Phosphorylation-dependent modulation of CFTR macromolecular signalling complex activity by cigarette smoke condensate in airway epithelia
Source: Sci Rep. 2019 Sep 3;9:12706. doi: 10.1038/s41598-019-48971-y (PMC6722123; doi:10.1038/s41598-019-48971-y)
Supplement: Supplementary file 1 — SUPPLEMENTARY INFO [file 41598_2019_48971_MOESM1_ESM.pdf]

SUPPLEMENTARY MATERIALS

Phosphorylation-dependent modulation of CFTR macromolecular signalling complex activity by cigarette smoke condensate in airway epithelia

Andrea Schnúr[1], Aiswarya Premchandar[1], Miklos Bagdany[1] and Gergely L. Lukacs[1,2,3]

[1]Department of Physiology, McGill University, Montréal, Quebec, Canada.

[2]Department of Biochemistry, McGill University, Montréal, Quebec, Canada.

[3]Groupe de Recherche Axé sur la Structure des Protéines (GRASP), McGill University, Montréal, Quebec, Canada.

Corresponding author:
Gergely L. Lukacs,
Department of Physiology
McGill University
3655 Sir-William-Osler
Montreal, QC, Canada   H3G 1Y6
e-mail: gergely.lukacs@mcgill.ca

SUPPLEMENTARY METHODS

Cell surface CFTR quantification

CFBE were treated (200 µg/ml CSC, DMSO, or 10 µM forskolin) in Krebs-Ringer Bicarbonate (KRB; 140 mM Na^+, 120 mM Cl^-, 5.2 mM K^+, 25 mM HCO_3^-, 2.4 mM HPO_4, 0.4 mM H_2PO_4, 1.2 mM Ca^{2+}, 1.2 mM Mg^{2+} and 5 mM glucose, pH 7.4) buffer for 30 min at 37°C. After treatment, the cells were washed twice with PBS++ (PBS containing 1 mM $MgCl_2$ and 0.1 mM $CaCl_2$) and blocked with PBS++-containing bovine serum albumin (0.5% BSA-PBS++) for 30 min. The primary anti-HA antibody and secondary HRP anti-mouse antibody (Jackson ImmunoResearch) incubation were done for an hour each on ice (1:1000 dilution), with four PBS++ washes between the two incubation steps. After eight washes with PBS++, the HRP activity was determined with the cell-impermeant Amplex Red HRP fluorogenic substrate (Invitrogen) at 544 nm excitation and 590 nm emission wavelengths using the TECAN fluorescence plate reader. Non-specific binding was determined using non-specific mouse IgG under the same experimental conditions.

For cell surface biotinylation, apical PM proteins of filter-grown cells were biotinylated on ice with 1 mg/ml EZ Link sulfo-NHS-SS-biotin (Thermo Fisher Scientific) for 15 min in buffer H (154 mM NaCl, 3 mM KCl, 10 mM Hepes, 1mM $MgCl_2$, 0.1 mM $CaCl_2$, 10 mM glucose, pH 7.8). Excess biotin reagent was washed off and quenched with 1% BSA-PBS++. Cells were then lysed in RIPA buffer (150 mM NaCl, 20 mM Tris-HCl, 1% Triton X-100, 0.1% SDS, and 0.5% sodium deoxycholate, pH 8.0) containing protease inhibitors (Leupeptin, Pepstatin A, phenylmethylsulfonyl fluoride). Biotinylated proteins were incubated with streptavidin-agarose beads (Invitrogen) or monomeric avidin magnetic beads (BcMag) at 4°C with rotation for an hour. Beads were washed three times with 1 ml RIPA buffer. Proteins were eluted from the beads with 5xLaemmli sample buffer (6.25% SDS, 250mM DTT, 150 mM Tris pH6.8, 20 mM EDTA, 0.875% glycerol, 0.017.5% BPB) supplemented with 6 mM biotin for 20 min at RT, separated by SDS-PAGE, and transferred onto a nitrocellulose membrane. Immunoblots were visualised with mouse anti-HA or Hsp90

antibodies and by HRP-conjugated anti-mouse antibodies (GE Healthcare). Densitometry analysis was performed with Image J.

Short circuit current measurement

CFBE and NCI-H441 were grown on fibronectin-coated 12 mm Snapwell filters (Corning) for at least four days post-confluency. Inserts were mounted in Ussing chambers, bathed in KRB, maintained at 37°C, and bubbled continuously with 95% O_2 and 5% CO_2. To functionally isolate the apical membrane, the basolateral side was permeabilised with amphotericin B (100 µM). To impose an apical-to-basolateral Cl gradient, NaCl and KCl in the apical bathing solution were replaced with Na^+ and K^+ gluconate, respectively. CR-HBE cells were seeded on Collagen IV-coated Snapwell filters, and during measurement, both apical and basolateral side was bathed in KRB. All measurements were performed under I_{sc} conditions recorded with the Acquire and Analyze package (Physiologic Instruments, San Diego, CA), and expressed as current/cm^2. In each experiment, amiloride (100 µM) and forskolin or CSC were added sequentially to the apical side followed by the addition of CFTR inhibitor (Inh-172; 20 µM) to demonstrate that measured currents were CFTR-specific. Each condition was measured in two technical replicates in every experiment. Transepithelial electrical resistance (TEER) was continuously monitored during the measurement and was >300 ohms*cm^2.

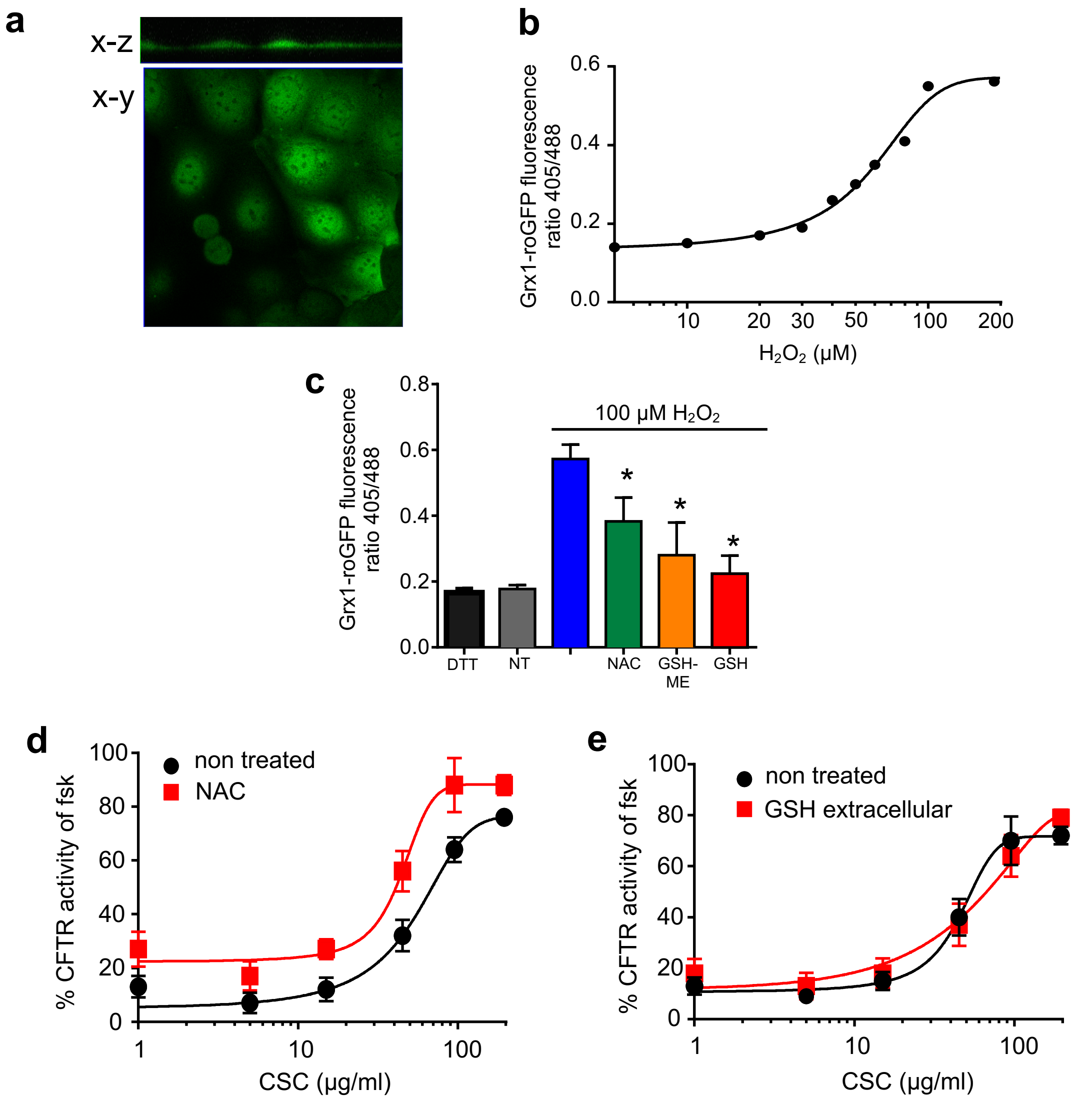

Supplementary Fig. 1. The impact of reducing agents on the CSC-stimulated CFTR activation in CFBE. a) Schematic representation and laser confocal images showing the cellular localization of human glutaredoxin-1 (Grx1)-fused redox-sensitive green fluorescent proteins (roGFP2) stably expressed in the inducible CFTR-expressing CFBE. Subcellular localization of the Grx1-roGFP vas visualized by fluorescence microscopy. **b)** Grx1-roGFP2 protein-expressing CFBE were grown in black 96-well plates 5-6 days post-confluency. Cells were treated with the cell permeable-reducing agent 500 μM DTT, or varying hydrogen peroxide concentrations (10, 20, 30, 50, 100, 200 μM H_2O_2), a cell-permeable oxidizing agent. After 10 min, the cells were excited at 405 and 488 nm and the ratio of fluorescence emission was calculated and plotted. Higher ratio value represents a higher oxidative state. Data were acquired using a fluorescence plate reader as described in Methods and show one representative measurement. **c)** The 405/488 nm ratio values without treatment (NT), after 20 min 500 μM DTT treatment (DTT) or 10 min treatment with 100 μM H_2O_2 with or without 4 h pre-treatment with 5 mM N-acetyl cysteine (NAC) or 1 mM glutathione monoethyl-ester (GSH-ME) or 10 min pre-treatment with 10 mM reduced L-glutathione (GSH). *p<0.05 n=3-6. **d)** Dose-response of CSC on CFBE with or without 4 h pre-treatment with 5mM NAC expressed as a percentage relative to maximal forskolin stimulation (254 ±20 μA/cm^2) **e)** Dose-response of CSC on CFBE with or without 10 min of pre-treatment with 10 mM reduced L-GSH expressed as a percentage relative to maximal forskolin stimulation (244 ±24 μA/cm^2).

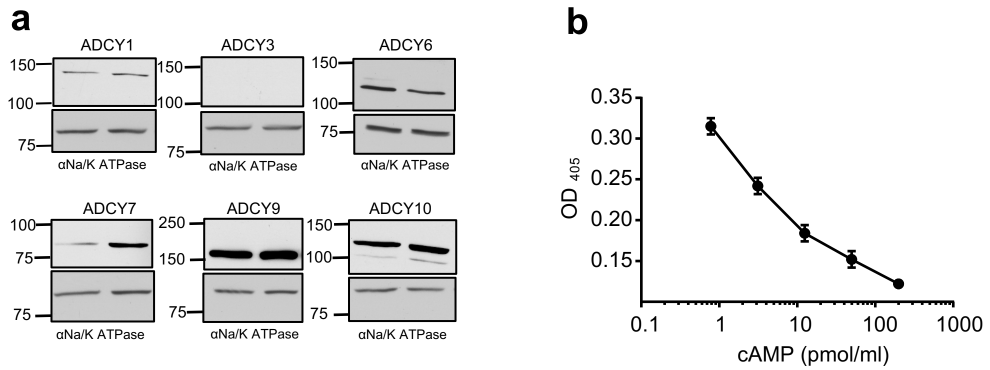

Supplementary Fig. 2. a) The expression pattern of adenylyl cyclase (ADCY) isoforms in CFBE. Immmunoblot analysis of whole cell lysates of CFBE was performed to probe the expression of ADCY1, 3,6,7,9 and10. The Na^+/K^+ ATPase was the loading control**. b) Calibration curve of the cAMP EIA assay.** Data are means ±SEM, n=4-7.

S422 –KTp**S**NGDDSLFFSNFSLLGTPVLKDINFK

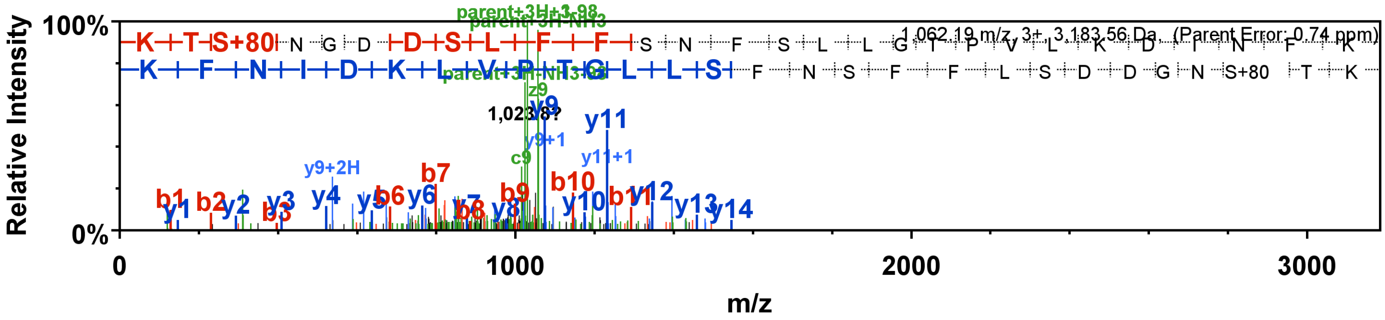

S660 –RNp**S**ILTETLHR

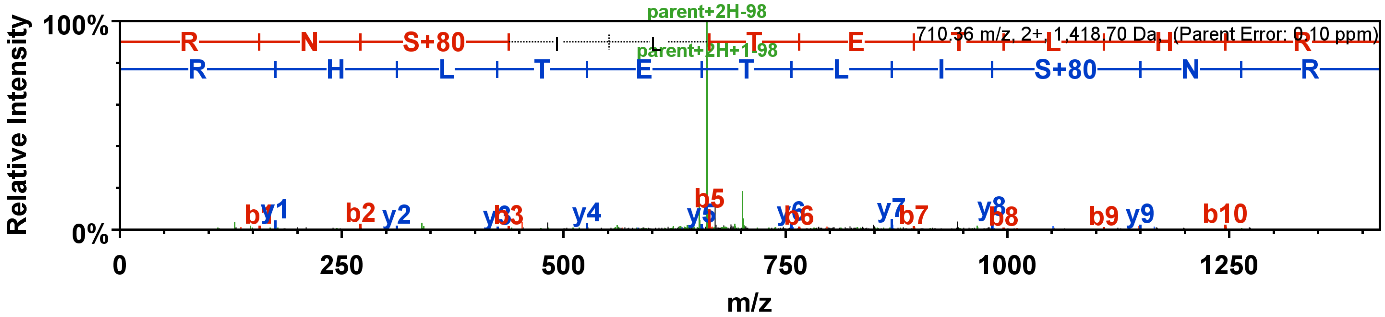

S670 –NSILTETLHRFp**S**LEGDAPVSWTETK

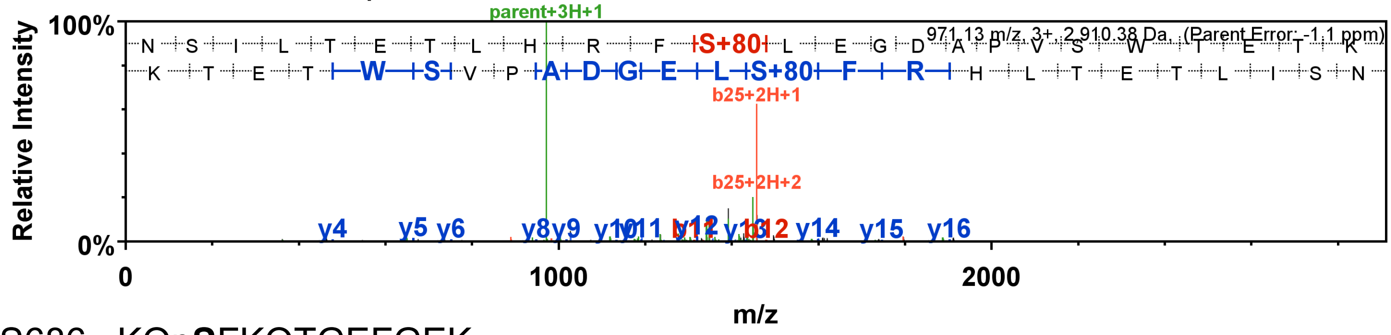

S686 –KQp**S**FKQTGEFGEK

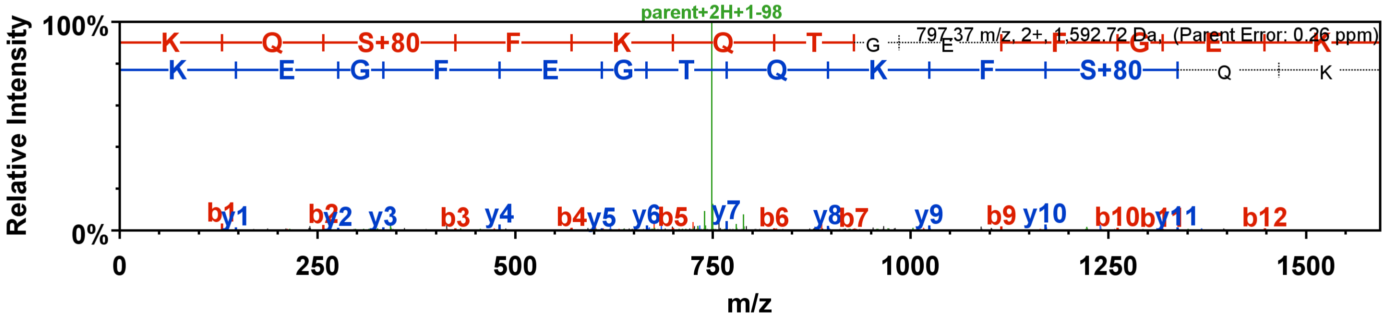

S700 –KNp**S**ILNPINSIR

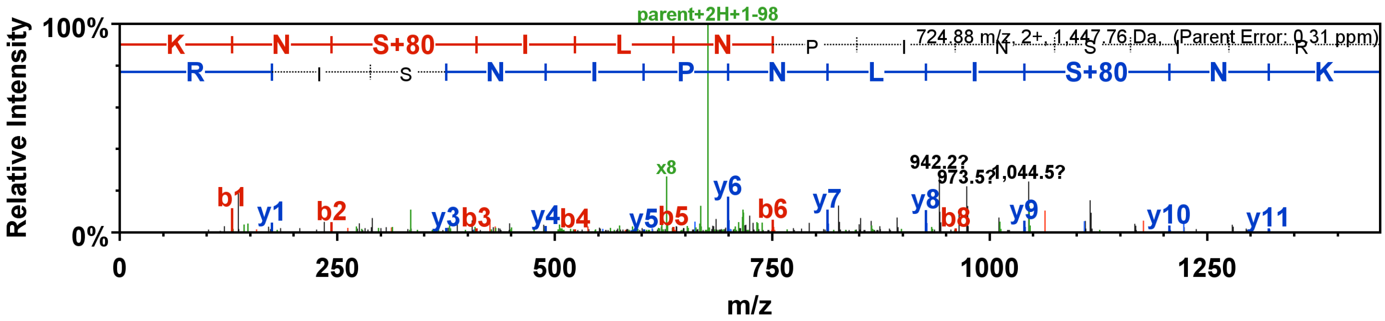

S712 –KFp**S**IVQK

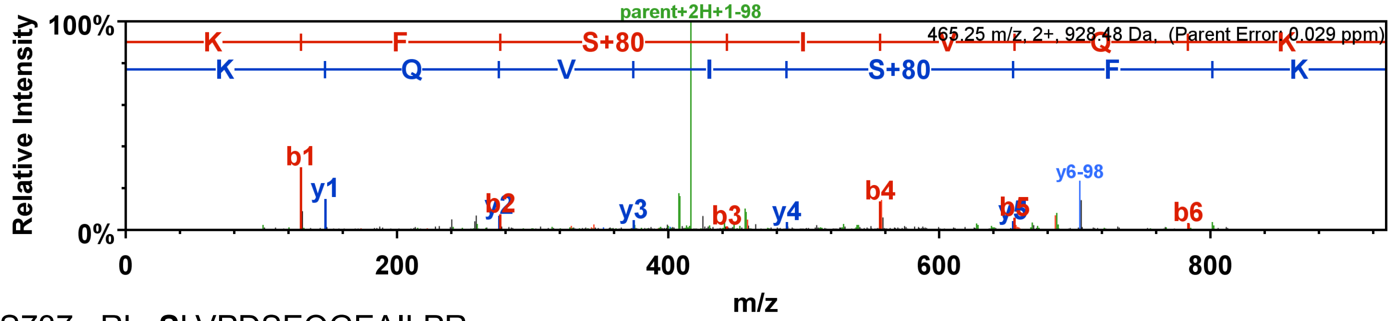

S737 –RLp**S**LVPDSEQGEAILPR

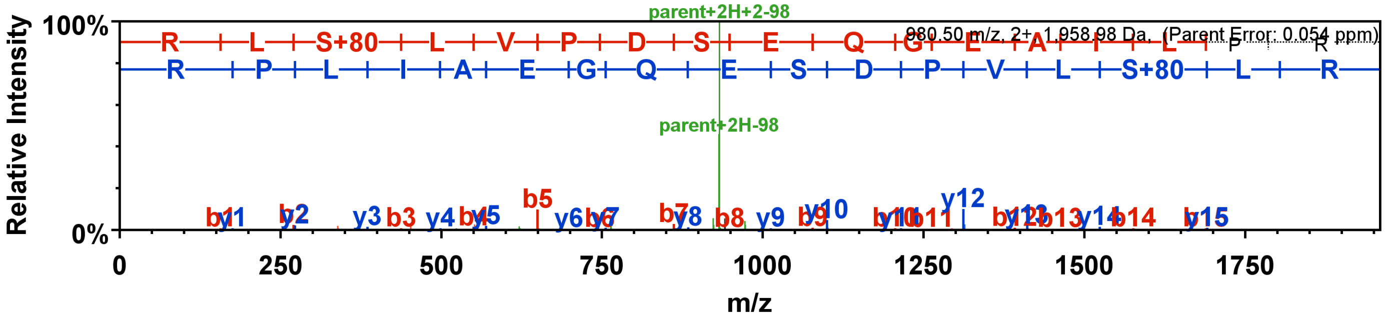

S753 –Ip**S**VISTGPTLQAR

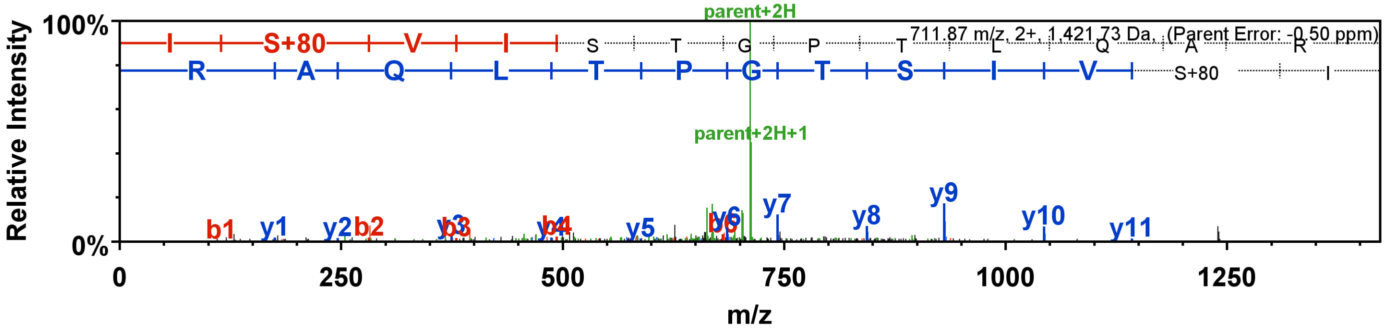

S768 –RQp**S**VLNLMTHSVNQGQNIHR

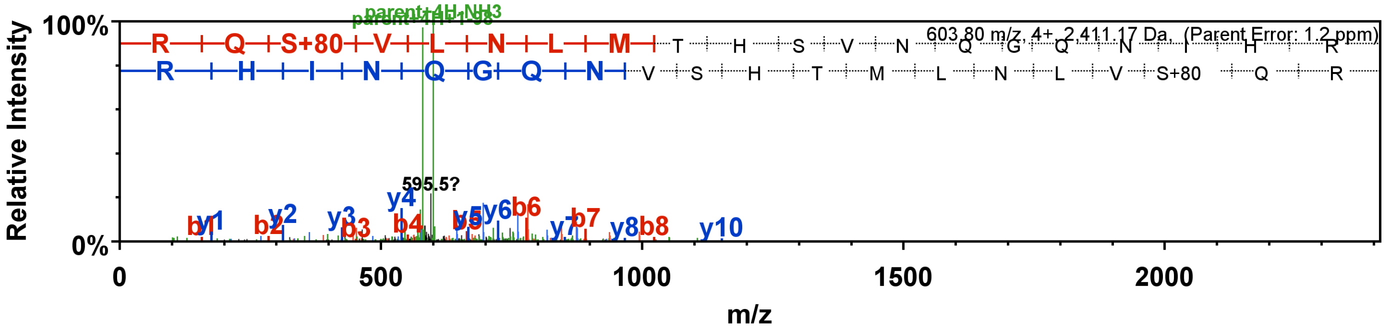

S795 –KVp**S**LAPQANLTELDIYSR

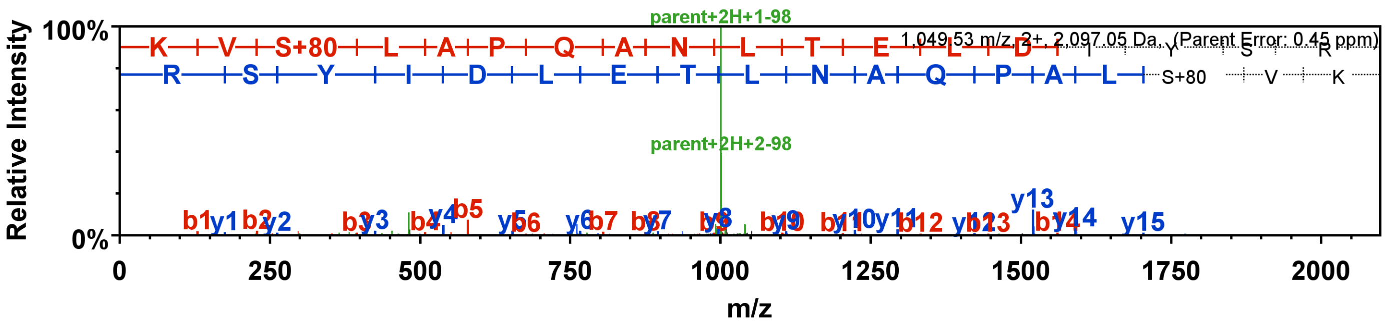

Supplementary Fig. 3.

S795 –KVp**S**LAPQANLTELDIYSR

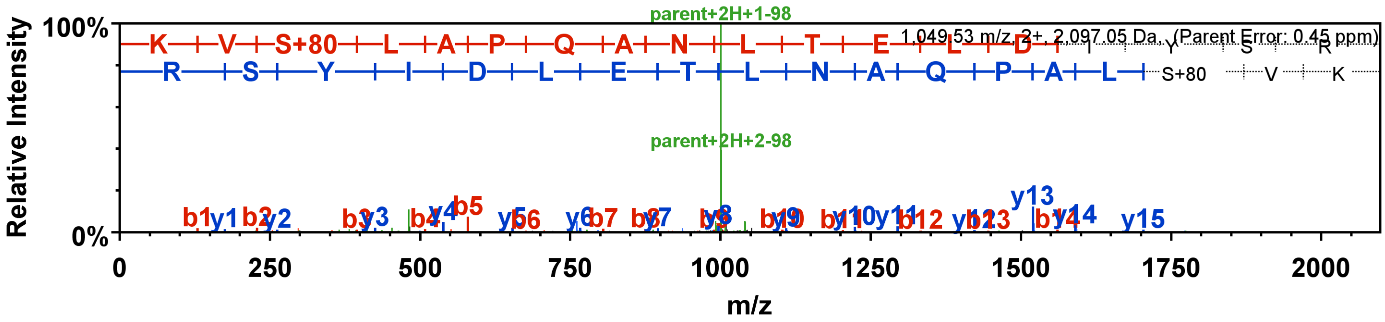

Supplementary Fig. 3. Manual validation of MS^n spectra using Scaffold software. The confidence of phosphosite assignment was manually validated for all the phosphosite-containing peptides. The individual peptide-associated spectra from a representative peptide corresponding to each phosphosite is shown here.

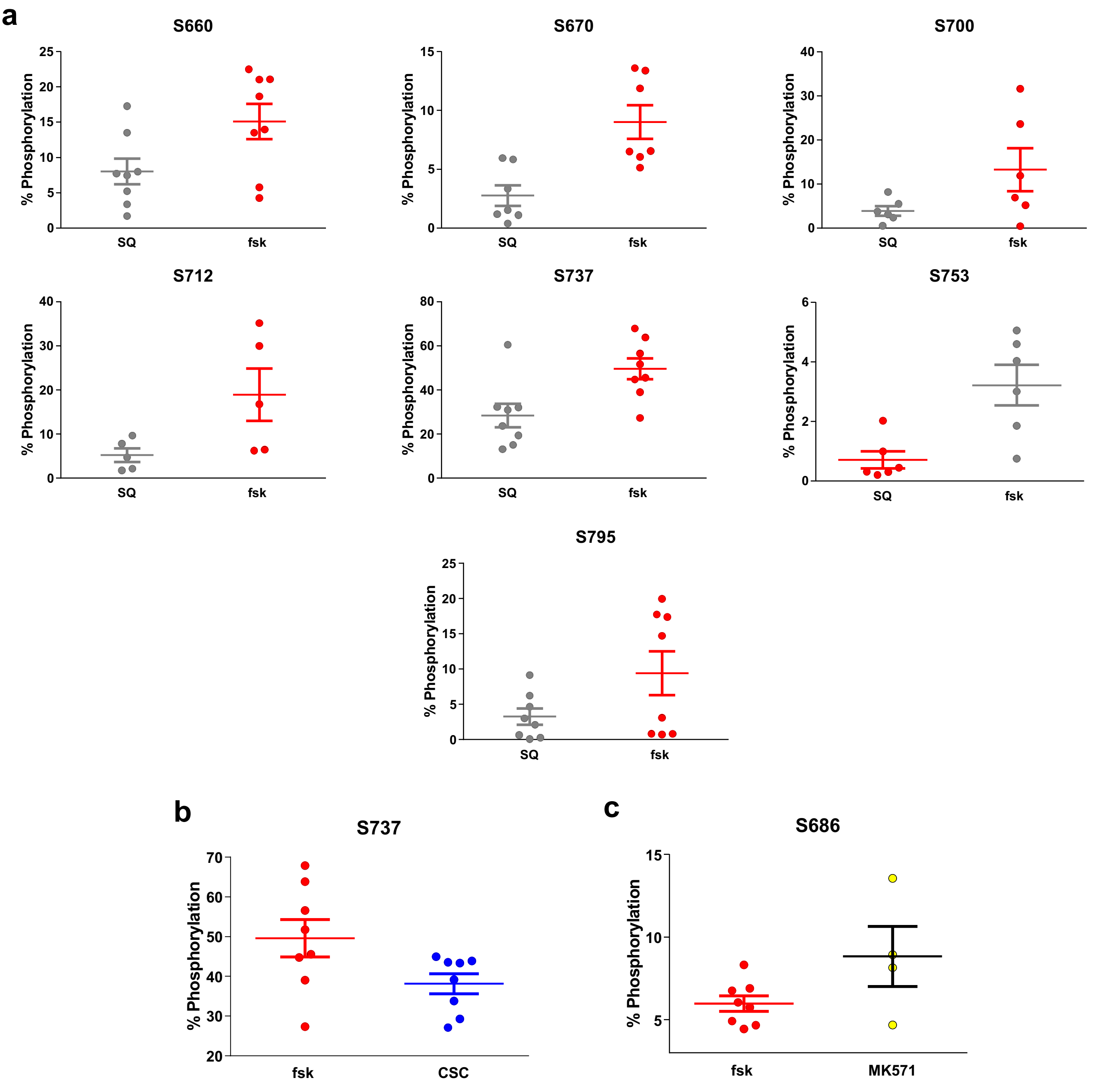

Supplementary Fig.4. Scatter plots of individual phosphosites that show a significant difference in phospho-occupancy between two treatment conditions. The horizontal line represents the mean of at least four biological replicates with the points representing the phospho-occupancy after 10 μM forskolin (a), 200μg/mL CSC(b), or 50 μM MK571 (c) exposure in each experiment. The error bars denote the standard error of the mean, n=5-8.

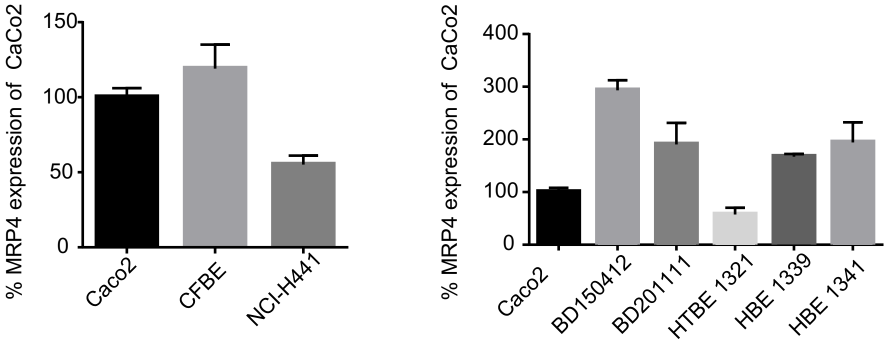

Supplementary Fig.5. MRP4 mRNA expression detection by qPCR. Quantitative PCR of MRP4 in immortalized airway epithelia (CFBE, NCI-H441) and CaCo-2 epithelia, as well as in primary CR-HBE$^{WT/WT}$ cells, obtained from 5 individuals. CaCo-2 epithelia was used as a reference for MRP4 transcript level.

Region of CFTR	Site	A-score (mean ± SEM)	*In vitro*	*In vivo*
NBD1	S422	19±3	25330774, 8880910	
RD	S660	61±2	1377674, 15657296, 1716180 25330774, 1716180, 8880910	9385646, 15657296, 25330774, 1716180, 22119790
	S670	36±4	15657296, 25330774	25330774
	S686	71±3		22119790
	S700	135±12	1377674, 15657296, 25330774, 1716180, 8880910	9385646, 15657296, 25330774, 22119790
	S712	1000±0	25330774, 1716180, 8880910	9385646, 15657296, 25330774, 22119790
	S737	117±2	1377674, 15657296, 1716180, 25330774, 8880910	9385646, 15657296, 25330774, 1716180, 22119790
	S753	61±9	15657296, 25330774	9385646
	S768	64±8	1377674, 15657296, 25330774, 1716180, 8880910	9385646, 15657296
	S795	169±3	1377674, 15657296, 1716180, 25330774, 8880910	9385646, 15657296, 25330774, 1716180, 22119790

Supplementary Table 1. Identified PKA-consensus sites in the RD and NBD1 of CFTR. The PMIDs of the publications that identified and characterised these sites either *in vitro* or *in vivo* are indicated.
